# A single-amino acid substitution in the adaptor LAT accelerates TCR proofreading kinetics and alters T-cell selection, maintenance and function

**DOI:** 10.1038/s41590-023-01444-x

**Published:** 2023-03-13

**Authors:** Wan-Lin Lo, Miriam Kuhlmann, Gabrielle Rizzuto, H. Atakan Ekiz, Elizabeth M. Kolawole, Monica P. Revelo, Rakieb Andargachew, Zhongmei Li, Yuan-Li Tsai, Alexander Marson, Brian D. Evavold, Dietmar Zehn, Arthur Weiss

**Affiliations:** 1 Division of Microbiology and Immunology, Department of Pathology, University of Utah School of Medicine, Salt Lake City, UT USA; 2 Division of Animal Physiology and Immunology, School of Life Sciences, Technical University of Munich, Freising, Germany; 3 Human Oncology and Pathogenesis Program, Department of Pathology and Laboratory Medicine, Memorial Sloan Kettering Cancer Center, New York, NY USA; 4 Department of Molecular Biology and Genetics, Izmir Institute of Technology, Gulbahce, Turkey; 5 Department of Pathology, University of Utah School of Medicine, Salt Lake City, UT USA; 6 Department of Microbiology and Immunology, University of California San Francisco, San Francisco, CA USA; 7 Division of Rheumatology, Rosalind Russell and Ephraim P. Engleman Arthritis Research Center, Department of Medicine, University of California San Francisco, San Francisco, CA USA; 8 Gladstone-UCSF Institute of Genomic Immunology, San Francisco, CA USA; 9 Chan Zuckerberg Biohub, San Francisco, CA USA; 10 Department of Medicine, University of California San Francisco, San Francisco, CA USA; 11 Diabetes Center, University of California San Francisco, San Francisco, CA USA; 12 Innovative Genomics Institute, University of California Berkeley, Berkeley, CA USA; 13 UCSF Helen Diller Family Comprehensive Cancer Center, University of California San Francisco, San Francisco, CA USA; 14 Parker Institute for Cancer Immunotherapy, University of California San Francisco, San Francisco, CA USA; 15 Institute for Human Genetics, University of California San Francisco, San Francisco, CA USA

**Keywords:** Signal transduction, Immune tolerance, Infection, T-cell receptor

## Abstract

Mature T cells must discriminate between brief interactions with self-peptides and prolonged binding to agonists. The kinetic proofreading model posits that certain T-cell antigen receptor signaling nodes serve as molecular timers to facilitate such discrimination. However, the physiological significance of this regulatory mechanism and the pathological consequences of disrupting it are unknown. Here we report that accelerating the normally slow phosphorylation of the linker for activation of T cells (LAT) residue Y136 by introducing an adjacent Gly135Asp alteration (LAT^G135D^) disrupts ligand discrimination in vivo. The enhanced self-reactivity of LAT^G135D^ T cells triggers excessive thymic negative selection and promotes T-cell anergy. During *Listeria* infection, LAT^G135D^ T cells expand more than wild-type counterparts in response to very weak stimuli but display an imbalance between effector and memory responses. Moreover, despite their enhanced engagement of central and peripheral tolerance mechanisms, mice bearing LAT^G135D^ show features associated with autoimmunity and immunopathology. Our data reveal the importance of kinetic proofreading in balancing tolerance and immunity.

## Abstract (web summary)

Lo and colleagues provide evidence for the TCR kinetic proofreading model by LAT Gly135Asp alteration to reveal functional consequences of altered kinetics in TCR activation in thymic selection and mature T-cell responses.

## Main

Adaptive T-cell immunity generates a highly diverse T-cell antigen receptor (TCR) repertoire for pathogen recognition that does not cause autoimmunity. The goal of TCR ligand discrimination is that agonist peptide bound to major histocompatibility complex (pMHC) triggers T-cell responses, whereas self-pMHC signals maintain T-cell survival^1,2^. Improper TCR ligand discrimination can cause autoimmunity and other immune-mediated diseases. Notably, TCR affinity for an agonist or self-pMHC may differ by only ten- to 15-fold^3^. In addition, TCR affinity for pMHC ligands is in the micromolar range, contrasting the binding affinities of B cell receptors, cytokine receptors and other receptor tyrosine kinases for their ligands, which are often in the nanomolar range^4^. These TCR characteristics make it difficult for T cells to reliably discriminate between self- and foreign pMHCs, to modulate the quality and quantity of resulting responses and to balance between immunity and tolerance. Several models have attempted to explain how T cells distinguish between self-peptides and foreign ligands, but in vivo evidence has been limited and the underlying mechanisms remain enigmatic.

The kinetic proofreading model suggests that a series of signaling events, some of which may include nodes that function as critical molecular timers, set an activation threshold for T cells^5–10^. In essence, a ligand must bind to the TCR for long enough to initiate a series of reversible kinetic proofreading events to be considered a bona fide TCR activation signal^5–10^. Typically, when a self-pMHC engages a TCR, the binding lifetime is too short to initiate all of the necessary proofreading events to activate the T cell, although it may induce responses that contribute to cell survival^1^. Consistent with the kinetic proofreading model, adaptation to intrinsic signaling events and modification of the signaling network (for example, upregulation of programmed cell death protein 1 (PD-1) or other negative regulators) can fine-tune the activation threshold in T cells. However, it is not known how a single, specific kinetic proofreading step can influence primary T-cell function in vivo. Addressing this question requires the identification of a bona fide kinetic proofreading step.

We previously identified the tyrosine residue Y136 in mammalian linker for activation of T cells (LAT) as a molecular timer that modulates TCR ligand discrimination in T cells in vitro^11^. LAT Y136 is the only tyrosine residue that, upon phosphorylation, is able to recruit phospholipase C-γ1 (PLC-γ1)^7,12,13^. Importantly, PLC-γ1 signaling cascades activate the transcription factor nuclear factor of activated T cells (NFAT), which regulates the expression of essential development and activation genes^14,15^. Abolishing LAT Y136-mediated signals perturbs naive T-cell homeostasis and tolerance^16–20^. The response patterns and frequency of calcium–NFAT signals also dictate T-cell responsiveness during immune responses; for example, persistent NFAT signals may lead to T-cell exhaustion^21,22^. In addition to activating PLC-γ1 downstream signaling cascades^9,10^, Y136 has two other unique features among known Zap-70 phosphorylation sites in LAT: (1) it has markedly slower phosphorylation kinetics in vitro than other Zap-70 targets; and (2) this is conferred by a glycine residue rather than an acidic residue preceding the substrate tyrosine^7,11,23^. Our recent data suggest that LAT Y136 phosphorylation constitutes an essential later kinetic proofreading step to support TCR ligand discrimination^11^. Moreover, they raise the question, ‘How does LAT Y136 phosphorylation-mediated kinetic proofreading contribute to T-cell fate determination in vivo?’.

In the current study, we reveal the physiological importance and pathological consequences of tuning the phosphorylation speed of LAT Y136. We generated a mouse model featuring T cells with altered kinetic proofreading by replacing Gly residue 135 with a negatively charged Asp residue (LAT^G135D^). In the thymus, LAT^G135D^ T cells are subjected to increased negative selection. In the periphery, the expression of LAT^G135D^ promotes specific phenotypic and functional adaptations, such as upregulation of CD5 and CD6 and induction of T-cell anergy. Strikingly, despite acquiring characteristics of enhanced tolerance in the thymus and in the periphery, LAT^G135D^ T cells retain augmented sensitivity and proliferative fitness in response to infection with *Listeria* strains expressing very weak ligands. However, LAT^G135D^ also promotes the terminal differentiation of antigen-specific CD8 T cells and impairs the formation of memory precursors in response to strong TCR stimuli. In addition, by 1 year of age, LAT^G135D^ female mice develop higher titers of autoantibodies than wild-type mice, along with signs of colitis. Our work therefore suggests that the rate of LAT Y136 phosphorylation relative to the TCR:pMHC binding lifetime is a critical parameter of TCR ligand discrimination and contributes to T-cell fate determination upon antigen encounter.

## Results

### Modifying T cells with altered TCR signal proofreading

**Extended Data Fig. 1:**
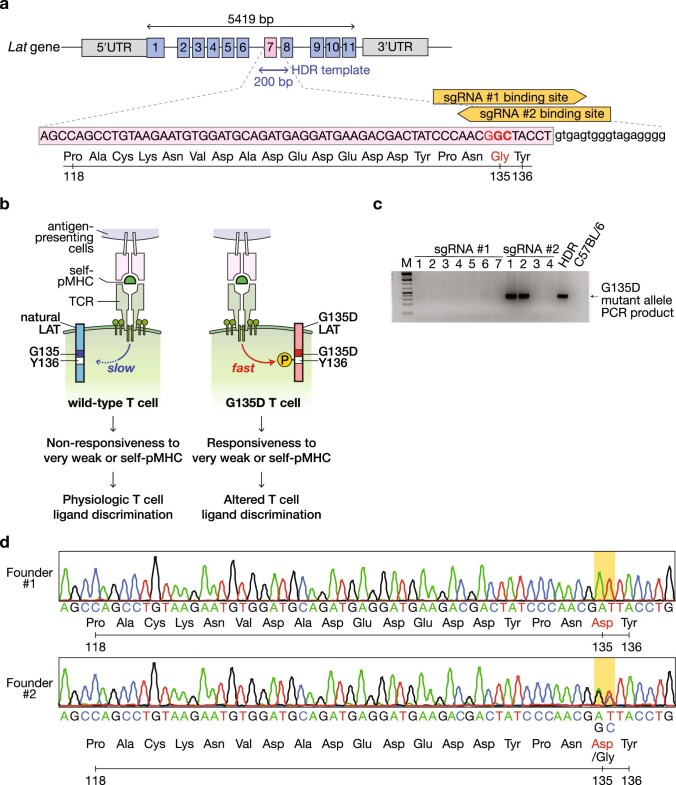
Generation of LAT G135D knock-in mouse. **a**. The cartoon illustrates the genomic locus of mouse *Lat*. The numbers represent individual exons. The nucleotide and amino acid sequences of exon 7 are depicted. The coding regions are listed in capital letters and the intron immediately following exon 7 is shown in lowercase letters. The orange arrows represent the two sgRNAs used to generate the G135D knock-in mice. **b**. Illustration of the molecular mechanisms underlying the engineering strategy. In brief, mammalian T cells express natural (wild-type) LAT, which exhibits slow phosphorylation kinetics upon TCR recognition of ligand; this serves as a proofreading bottleneck to create the molecular time delay required for proper TCR ligand discrimination. Only bona fide activating ligands that interact with the TCR with a sufficiently long bond lifetime pass this slow signaling bottleneck to activate T cells (left). On the other hand, the bond lifetime of an interaction between the TCR and self-pMHC is too short to activate T cells (left). Importantly, the phosphorylation of LAT Y136 is regulated by the amino acid preceding Y136. Natural LAT has a small glycine at the −1 position, leading to slow phosphorylation (right); sequence-modifying LAT with a negatively charged aspartate substantially facilitates the rate and magnitude of Y136 phosphorylation (right). G135D mutant LAT therefore bestows on T cells the ability to respond to very weak ligands or self-peptides. **c**. The PCR genotyping results of the seven pups born to the founder generated with CRISPR/Cas9 using sgRNA #1 and the four pups born to the founder generated with sgRNA #2. Homology-directed repair (HDR) initiated by electroporation provided the repair template for CRISPR/Cas9 and was used as a positive control for PCR screening. gDNA from C57BL/6 parents was used as a negative control. The numbers along the top of the gel represent the individual pups. Pups #1 and #2 from the sgRNA #2 experiments are founders #1 and #2, respectively. The genotyping result was performed once but confirmed with Sanger sequencing analysis. **d**. Four-color chromatograms of the exon 7 gDNA sequence analyses of founders #1 and #2.

To elucidate the consequences of disrupting a single bona fide kinetic proofreading step in an otherwise intact biological system, we utilized CRISPR–Cas9 technology to introduce a mutation into the endogenous mouse *Lat* locus that would be transcribed as the Gly135Asp alteration (Extended Data Fig. 1a–d). In LAT^G135D^ mice, the kinetics of a single proofreading signaling step (that is, phosphorylation of LAT Y136, which influences PLC-γ1 recruitment, phosphorylation and activation) is accelerated, endowing T cells with the ability to respond to weak or self-ligands in vitro (Extended Data Fig. 1b).

### Expression of G135D LAT alters thymocyte development

Immature thymocytes require TCR signals of appropriate strength to complete thymic development. Since the thymic selection thresholds are differentially regulated in neonates and adults^24^, we analyzed polyclonal thymocyte development in LAT^G135D^ knockin mice or wild-type mice at the neonatal (10- to 14-day-old) and adult (6-week-old) stages.

**Fig. 1:**
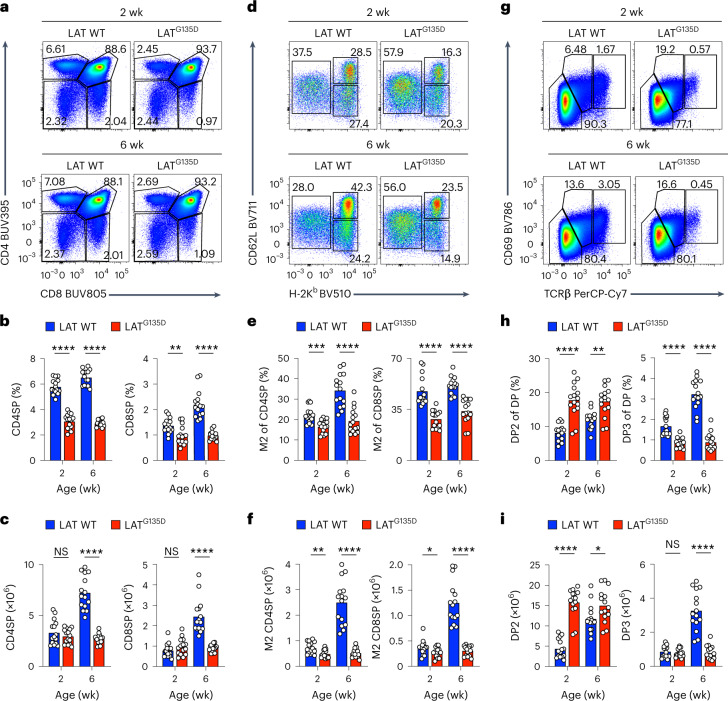
LAT^G135D^ affects thymopoiesis and decreases the production of single-positive thymocytes. **a**–**i**, Cellularity of thymi harvested from wild-type (WT) or LAT^G135D^ C57BL/6 mice as neonates (2 weeks old) or as adults (6 weeks old). The data are representative of at least four independent experiments. wk, weeks. **a**, Representative pseudocolor plots depicting the expression of CD4 and CD8. **b**,**c**, Bar graphs summarizing the percentages (**b**) and absolute numbers (**c**) of CD4SP (left) and CD8SP cells (right) among live thymocytes. **d**, Expression of the thymocyte maturation markers CD62L and MHC-I H-2K^b^ on CD4SP cells, including semi-mature (CD62L^–^H-2K^b–^; SM), mature stage 1 (CD62L^–^H-2K^b+^; M1) and mature stage 2 cells (CD62L^+^H-2K^b+^; M2). **e**,**f**, Bar graphs summarizing the percentages (**e**) and absolute numbers (**f**) of CD62L^+^H-2K^b+^ M2 CD4SP (left) and M2 CD8SP thymocytes (right). **g**, Representative pseudocolor plots showing CD69 and TCRβ expression profiles of DP thymocytes, including preselection DP1 (CD69^–^TCRβ^–^), midselection DP2 (CD69^med^TCRβ^med^) and postselection DP3 cells (CD69^hi^TCRβ^hi^). **h**,**i**, Bar graphs summarizing the percentages (**h**) and absolute numbers (**i**) of DP2 (left) and DP3 cells (right) among DP thymocytes. In **b**, **c**, **e**, **f**, **h** and **i**, each dot represents an individual mouse (*n* = 15). In **b**, **P = 0.0041 and ****P < 0.0001. In **c**, ****P < 0.0001, NS (not significant) = 0.5393 (left) and NS = 0.2169 (right). In **e**, ***P = 0.0001 and ****P < 0.0001. In **f**, ****P < 0.0001, **P = 0.0057 and *P = 0.0295. In **h**, **P = 0.0012 and ****P < 0.0001. In **i**, ****P < 0.0001, *P = 0.0209 and NS = 0.6236. Statistical significance was determined by two-tailed Mann–Whitney *U*-test. Source data

**Extended Data Fig. 2:**
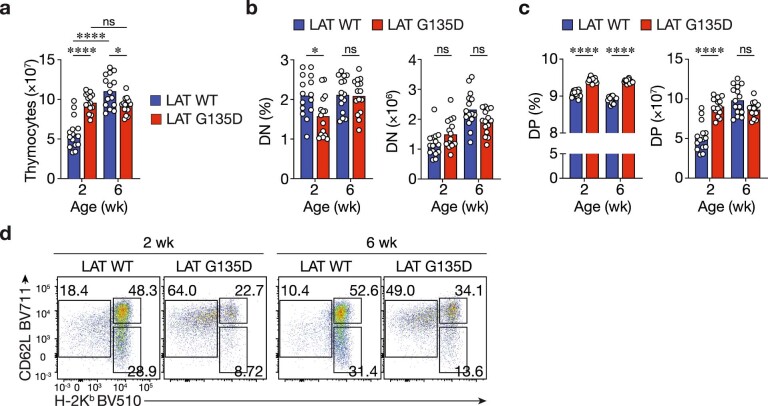
The thymic cellularity of G135D knock-in mice. **a**. Bar graphs show the total thymocyte numbers of mice at the indicated ages. wk: weeks. Bar graphs show the frequency (left) or absolute number (right) of double-negative (DN) thymocytes. **c**. Bar graphs show the frequency (left) or absolute number (right) of double-positive (DP) thymocytes. **d**. Representative flow plots of the expression of thymocyte maturation markers CD62L and MHC-I H-2K^b^ on CD8SP. **a–c**. Each dot represents an individual mouse. n = 15. Two-tailed Mann-Whitney test. *P = 0.0204, ****P < 0.0001, ns = 0.2901 (**a**); *P = 0.0111, ns = 0.8702 (left), ns = 0.0555 (middle), and ns = 0.0502 (right) (**b**); ****P < 0.0001 and ns = 0.0502 (**c**). Two-tailed Mann-Whitney test. Source data

During the postnatal period, thymus size progressively increased in the wild-type mice, almost doubling between the neonatal and adult stages. In LAT^G135D^ mice, the thymus was already enlarged at 2 weeks (Extended Data Fig. 2a). We observed less impact on the double-negative population (Extended Data Fig. 2b) but observed an effect on the double-positive (DP) and single-positive populations in LAT^G135D^ mice (Fig. 1a–c and Extended Data Fig. 2c). In adult LAT^G135D^ knockin mice, there were approximately 60% fewer single-positive CD4 (CD4SP) and single-positive CD8 (CD8SP) cells than in their wild-type littermates (Fig. 1a–c). Whereas the percentages of neonatal single-positive cells were also lower in LAT^G135D^ mice (Fig. 1a–c), the absolute numbers remained comparable to those in neonate wild-type littermates (Fig. 1c). Notably, among the LAT^G135D^ single-positive cells, the mature CD62L^+^H-2K^b+^ thymocytes ready for thymic egress were the most substantially affected population (Fig. 1d–f and Extended Data Fig. 2d) in mice of all ages.

To further investigate how altering the LAT Y136 kinetic proofreading step affected thymocyte development, we analyzed the DP thymocyte populations. DP cells gradually upregulate the expression of the TCR and the activation marker CD69 upon receipt of selecting signals, progressing from preselection DP1 (CD69^–^TCR^–^) to midselection DP2 (CD69^med^TCR^med^) to postselection DP3 (CD69^hi^TCR^hi^) thymocytes. The expression of LAT^G135D^ resulted in significantly lower frequencies and absolute numbers of DP3 cells in adult mice (Fig. 1g–i), which suggests that the Gly135Asp-induced defects in the CD4SP and CD8SP populations occurred at the DP2-to-DP3 thymocyte transition. Taken together, the data reveal that the expression of LAT^G135D^ resulted in substantially smaller CD4SP and CD8SP thymocyte populations in LAT^G135D^ mice and that immature adult thymocytes are more sensitive to LAT^G135D^-promoted signaling than neonatal cells.

### LAT^G135D^ expression triggers negative selection

**Fig. 2:**
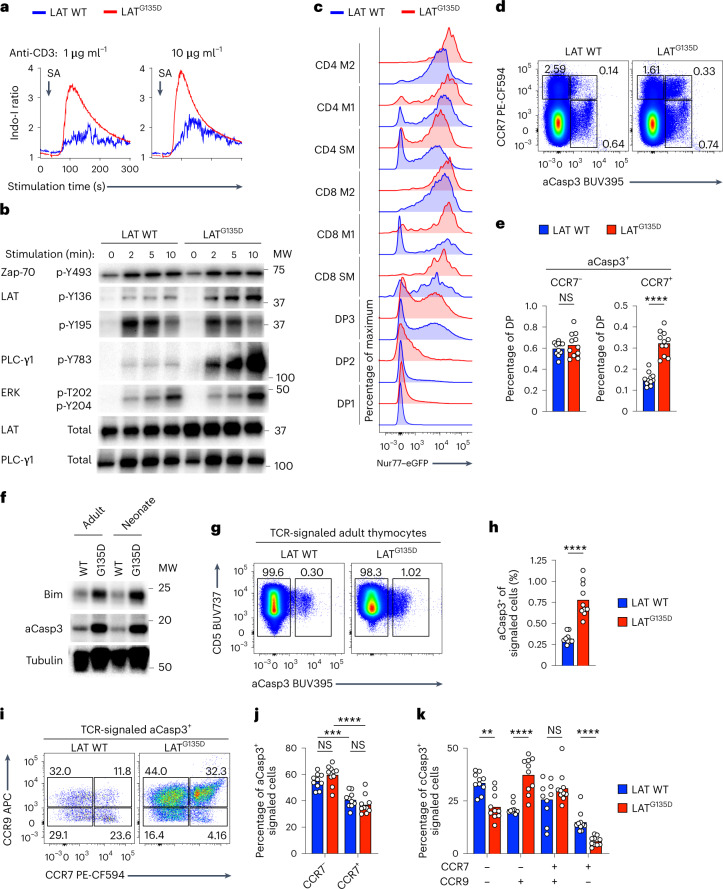
LAT^G135D^ promotes negative selection in the medulla. **a**, Representative calcium traces of wild-type and LAT^G135D^ CD53^–^ preselection DP thymocytes were analyzed before and after the addition of streptavidin (SA) to crosslink anti-CD3ε. **b**, Immunoblot analysis of specific proximal signaling proteins of wild-type or LAT^G135D^ CD53^–^ preselection DP thymocytes after crosslinking with anti-CD3ε antibody. MW, molecular weight of protein ladders (kDa). The two columns of labels on the left represent the protein name and the amino acid residue, respectively. The ‘p-’ indicates phosphorylation. **c**, Histogram of the expression of eGFP in various thymocyte developmental subsets from wild-type and LAT^G135D^ Nur77–eGFP reporter mice. **d**, Pseudocolor plots of the expression of CCR7 and cleaved caspase-3 (aCasp3) in DP thymocytes. **e**, Bar graphs summarizing the percentages of DP thymocytes undergoing apoptosis in the cortex (CCR7^–^aCasp3^+^) or ready to migrate to the medulla (CCR7^+^aCasp3^+^). Each dot represents a single mouse (*n* = 11). ****P < 0.0001 and NS = 0.5726. **f**, Immunoblot analysis of the total protein expression of Bim and aCasp3 of sorted wild-type and LAT^G135D^ DP thymocytes. **g**,**h**, Analysis of the expression of CD5 and aCasp3 on total thymocytes (CD5^+^TCRβ^+^; an example of the gating strategy is shown in Extended Data Fig. 3e). Representative pseudocolor plots (**g**) and summarized bar graphs (**h**) are shown. The numbers in **g** show the percentages of apoptotic (aCasp3^+^) and nonapoptotic (aCasp3^–^) cells. Each symbol in **h** represents a single mouse (*n* = 10). ****P < 0.0001. **i**–**k**, Representative pseudocolor plots (**i**) showing the expression of CCR9 and CCR7 on CD5^+^TCRβ^+^aCasp3^+^ thymocytes. The bar graphs show the percentages of CCR7^–^ and CCR7^+^ CD5^+^TCRβ^+^aCasp3^+^ thymocytes, indicative of clonal deletion in the cortex (CCR7^–^) versus cells ready to migrate to the medulla (CCR7^+^) (**j**) and stage of development (CCR9^+^CCR7^–^, CCR9^+^CCR7^+^ or CCR9^–^CCR7^–^; **k**). Each dot represents an individual mouse (*n* = 10). **P = 0.0011, ***P = 0.0001, ****P < 0.0001, NS = 0.0892 (**j**, left), NS = 0.1230 (**j**, right) and NS = 0.3930 (**k**). In **a**–**k**, the data are representative of two (**f**), three (**a**,**c**,**i**–**k**) or four (**b**,**d**,**e**,**g**,**h**) independent experiments. Statistical significance was determined by two-tailed Mann–Whitney *U*-test. Source data

**Extended Data Fig. 3:**
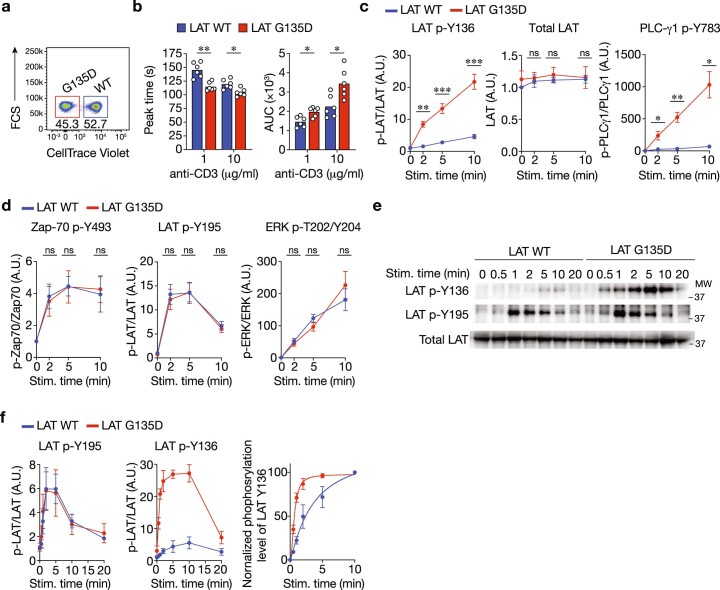
The expression of G135D augments LAT Y136–PLC-γ1 signaling. **a**. Representative flow cytometry plot of wild-type or G135D pre-selection CD53^–^ thymocytes labeled with titrated amounts of CellTrace Violet dye. FSC, forward scatter. **b**. Bar graphs depict the statistical analysis of calcium responses (Fig. 2a) of wild-type or G135D pre-selection CD53^–^ thymocytes in response to anti-CD3ε crosslinking (concentration as indicated). The bar graph at left shows the response time to reach the peak (unit: seconds, s). The bar graphs at the right show the area under curve (AUC) of the calcium responses; the larger the AUC, the stronger the calcium responses. **P = 0.0022 (left), *P = 0.0108 (middle left), *P = 0.0173 (middle right), *P = 0.0152 (right).. Two-tailed Mann-Whitney test. **c,d**. The band densities of phosphorylated LAT Y136 (**c**), PLC-γ1 (**c**), Zap-70 Y493 (**d**), LAT Y195 (**d**), ERK T202/Y204 (**d**) are shown. Data were standardized to the band density of the wild-type no-stimulatory control. Quantified data are summarized from six independent experiments. Error bars represent SEM. LAT p-Y136: ***P = 0.000778 (left), ***P = 0.000329, **P = 0.00127; Total LAT: ns = 0.781024 (middle left), ns = 0.514455 (middle right); ns = 0.852218 (right); PLC-γ1 Y783: **P = 0.001166 (right); *P = 0.005454 (right), *P = 0.023421 (left); Zap-70 p-Y783: ns = 0.663221 (middle left); ns = 0.966048 (middle right); ns = 0.708332 (right); LAT p-Y195: ns = 0.055783 (middle left), ns = 0.898515 (middle right); ns = 0.054248 (right); ERK p-T202/Y204: ns = 0.614684 (middle left), ns = 0.165792 (middle right), ns = 0.091339 (right). Paired student *t* test (Two sided).. A.U., arbitrary unit. **e,f**. Immunoblot analysis of wild-type or G135D LAT naive CD4 T cells after crosslinking with 5 μg/ml biotinylated anti-CD3ε antibody at 37 °C (time as indicated above the blots). Phosphorylation of total LAT or phopsho-LAT p-Y136 and p-Y195, was analyzed as indicated. The band densities of phosphorylated LAT Y136 and LAT Y195 are shown in the graphs in (**f, left and middle**). Normalized percentage of LAT Y136 phosphorylation level in relative to the maximum (**f, right**). Data are representative of four independent experiments. MW: molecular weight of protein ladders (unit: kDa). Data are presented as mean values ± SEM. Source data

To establish the cause of the smaller single-positive populations as defective positive selection, disrupted negative selection or death by neglect, we characterized the modifications in TCR signaling conferred by the Gly135Asp alteration. LAT^G135D^ or wild-type preselection CD53^–^ thymocytes were isolated ex vivo and labeled with different dilutions of CellTrace Violet (Extended Data Fig. 3a). The cells were mixed and then stimulated with crosslinking anti-CD3ε antibodies. LAT^G135D^ preselection cells exhibited a more rapid and much larger increase in cytoplasmic free calcium than that observed in wild-type preselection cells (Fig. 2a and Extended Data Fig. 3b). In contrast, wild-type preselection DP cells showed a slower, more sustained calcium increase (Fig. 2a and Extended Data Fig. 3b). Immunoblot analysis of such ex vivo-stimulated thymocytes further demonstrated that the expression of LAT^G135D^ led to enhanced phosphorylation of LAT Y136 and PLC-γ1 in preselection CD53^–^ thymocytes (Fig. 2b and Extended Data Fig. 3c). Importantly, activation of the kinase Zap-70 (as evidenced by phosphorylation of Y493 in its activation loop) and phosphorylation of other LAT tyrosine residues (such as Y195) in LAT^G135D^ thymocytes were comparable to levels in wild-type thymocytes (Fig. 2b and Extended Data Fig. 3d). Similar results were observed in peripheral naive CD4 T cells (Extended Data Fig. 3e,f). These results suggest that the Gly135Asp alteration selectively increases the phosphorylation speed and magnitude of Y136 and PLC-γ1.

**Extended Data Fig. 4:**
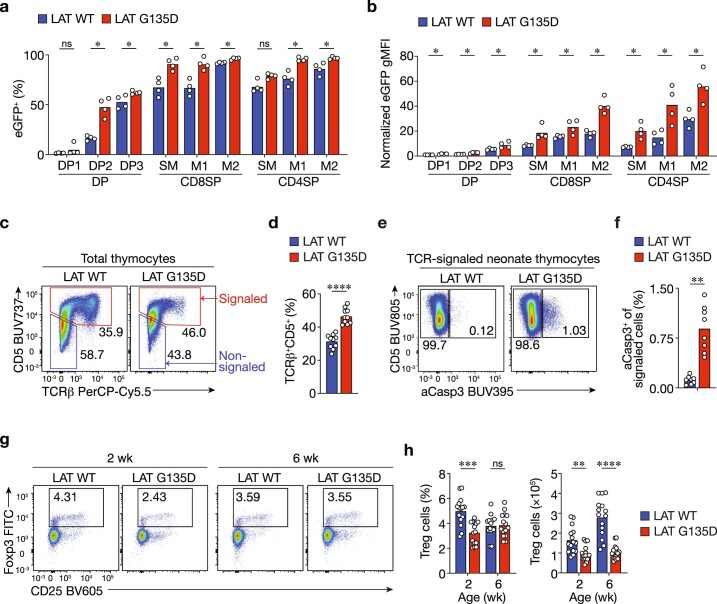
The expression of G135D alters thymic selection. **a,b**. Bar graphs summarize the percentage of Nur77-eGFP^+^ wild-type or G135D thymocytes among the indicated developmental subsets. Each symbol represents one individual mouse; n = 4; *P = 0.0286; ns = 0.571. Two-tailed Mann-Whitney test. **c,d**. Representative flow plots show the gating strategy for “signaled” or “non-signaled” wild-type or G135D LAT thymocytes (**c**). The signaled thymocytes are defined as the CD5^+^TCR^+^ population. The bar graph represents the frequency of “signaled” thymocytes (**d**). Each symbol represents one individual mouse; n = 10; ****P < 0.0001. Two-tailed Mann-Whitney test. **e,f**. Expression of CD5 and cleaved caspase-3 (aCasp3) in signaled thymocytes harvested from wild-type or G135D neonates. Each symbol represents one individual mouse; n = 8; **P = 0.0002. Two-tailed Mann-Whitney test. **g,h**. Representative flow plots (**g**) and bar graphs (**h**) show the expression of Foxp3 and CD25. Each symbol represents one individual mouse; n = 15. **P = 0.0020; ***P = 0.0001; ****P < 0.0001; ns = 0.8943. Two-tailed Mann-Whitney test. wk: weeks. Source data

Next, we investigated TCR signaling in LAT^G135D^ mice following physiologically relevant positive selection stimulation in the thymus^25–27^. Consistent with the in vitro biochemical findings (Fig. 2b), the LAT^G135D^ T cells demonstrated stronger orphan nuclear hormone receptor Nur77 activation in vivo (Fig. 2c), probably due to encountered self-pMHCs, as indicated by flow cytometric analysis of LAT^G135D^ Nur77–enhanced green fluorescent protein (eGFP) reporter bacterial artificial chromosome transgenic mice^28^. Notably, there were more eGFP^+^ cells among post-DP2 LAT^G135D^ thymocytes than among corresponding cells from wild-type littermates (Fig. 2c and Extended Data Fig. 4a) and the geometric mean fluorescence intensity of eGFP was also higher in LAT^G135D^ thymocytes than in wild-type cells (Fig. 2c and Extended Data Fig. 4b). It was particularly noteworthy that twice as many LAT^G135D^ compared with wild-type DP cells displayed a cleaved caspase-3^+^ (aCasp3^+^) and chemokine receptor CCR7^+^ phenotype, consistent with ongoing apoptosis due to clonal deletion of thymocytes migrating to the medulla^25,29–31^ (Fig. 2d,e). Immunoblot analysis of both adult and neonatal LAT^G135D^ DP thymocytes compared with wild-type counterparts further confirmed elevated expression of aCasp3 and proapoptotic Bcl-2 family member Bim (Fig. 2f).

Since clonal deletion can occur throughout the maturation process, we furthered assessed the clonal deletion of total thymocytes that received TCR signals^31^ (Extended Data Fig. 4c). Among thymocytes that had experienced TCR signals based on CD5 upregulation^31^ (Extended Data Fig. 4d), there was a threefold increase in the aCasp3^+^ population in the adult LAT^G135D^ mice compared with that in wild-type littermates (Fig. 2g,h) and an eightfold increase in LAT^G135D^ neonates (Extended Data Fig. 4e,f). We measured the expression levels of CCR9 and CCR7 on TCR-signaled aCasp3^+^ thymocytes to approximate the effects of the alteration on the anatomic location and timing of clonal deletion. For both wild-type and LAT^G135D^ thymocytes, roughly 60% of clonal deletion occurred in the cortex (Fig. 2i,j), consistent with previous reports^25,31,32^. Interestingly, in comparison with wild-type thymocytes, a larger proportion of LAT^G135D^ thymocytes underwent clonal deletion at the semi-mature, proliferation-incompetent CCR9^+^ stage (Fig. 2i,k), which may explain the altered maturation pattern in LAT^G135D^ mice (Fig. 1c,d). In addition, clonal deletion during CD4SP maturation usually correlates with failed regulatory T-cell (T_reg_ cell) development^1^. We observed fewer T_reg_ cells in LAT^G135D^ mice (Extended Data Fig. 4g,h). These results indicate that the expression of LAT^G135D^ promotes negative selection, possibly due to enhanced thymocyte reactivity to self-pMHC stimuli caused by augmented TCR-dependent LAT Y136–PLC-γ1 signaling.

### LAT^G135D^ promotes homeostatic proliferation

**Fig. 3:**
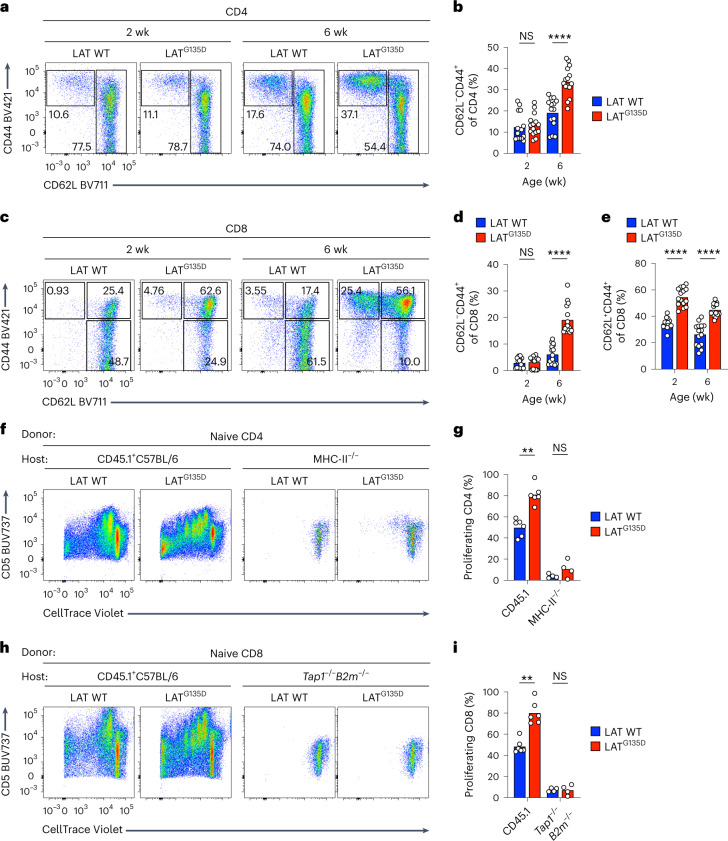
LAT^G135D^ augments self-peptide-driven homeostatic proliferation of peripheral T cells. **a**,**c**, Representative pseudocolor plots of the expression of CD62L and CD44 on peripheral spleen CD4 (**a**) and CD8 T cells (**c**) from wild-type versus LAT^G135D^ neonatal (2 week) and adult (6 week) mice. The numbers associated with the gates show the percentages of naive (CD62L^+^CD44^–^), central memory (CD62L^+^CD44^+^) and effector memory (CD62L^–^CD44^+^) cells. **b**,**d**,**e**, Bar graphs depicting the percentages of effector memory (CD62L^–^CD44^+^) cells among peripheral CD4 (**b**) and CD8 (**d**) T cells and the percentages of central memory (CD62L^+^CD44^+^) cells among peripheral CD8 T cells (**e**). Each dot represents a single mouse (*n* = 15). The data are representative of at least five independent experiments. ****P < 0.0001, NS = 0.4302 (**b**) and NS = 0.9588 (**d**). **f**–**i**, Naive CD4 (**f**,**g**) or CD8 (**h**,**i**) T cells were sorted from 4- to 5-week-old wild-type or LAT^G135D^ mice, labeled with CellTrace Violet and adoptively transferred intravenously into congenic hosts (CD45.1^+^), MHC-II^–/–^ hosts or *Tap1*^–/–^*B2m*^–/–^ hosts (as indicated) that had been sublethally irradiated (300 rads) the day before. The dilution of CellTrace Violet was assessed by flow cytometry 4 d post-transfer. **f**,**h**, Representative flow plots of CellTrace Violet dilution and the expression of CD5. **g**,**i**, Bar graphs summarizing the percentages of adoptively transferred CD4 (**g**) and CD8 cells (**i**) that underwent proliferation. Each dot represents an individual mouse (*n* = 6 for the CD45.1^+^ C57BL/6 host and *n* = 4 for the MHC-II^–/–^ and *Tap1*^–/–^*B2m*^–/–^ hosts. The data were compiled from three independent experiments. **P = 0.0043 (**g**), **P = 0.0022 (**i**), NS = 0.3429 (**g**) and NS = 0.9429 (**i**). Statistical significance in **b**, **d**, **e**, **g** and **i** was determined by two-tailed Mann–Whitney *U*-test. Source data

**Extended Data Fig. 5:**
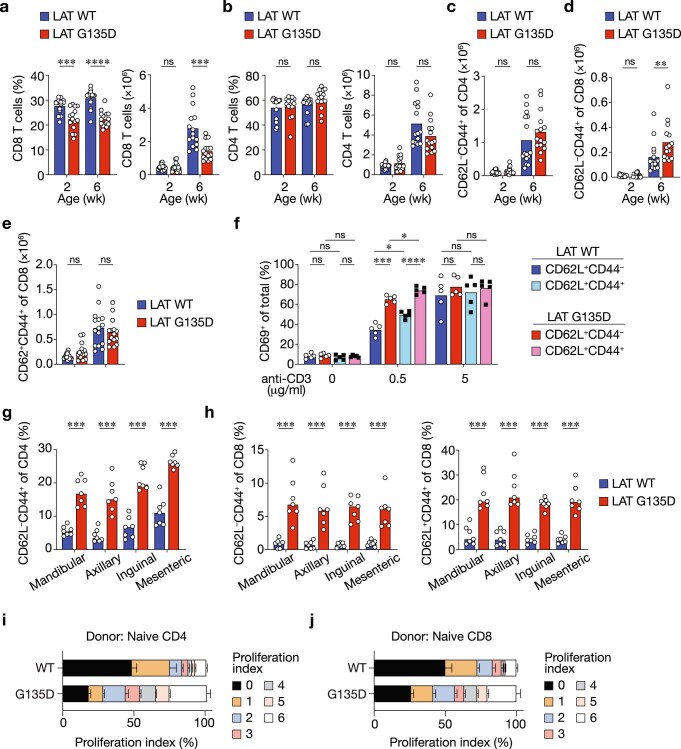
The expression of G135D in peripheral T cells promotes the emergence of “memory-phenotype” T cells. **a,b**. Bar graphs show the percent (left) or absolute number (right) of CD8 (**a**) or CD4 (**b**) T cells in the periphery at the indicated ages. wk: weeks. Data are representative of 4 independent results. **c–e**. Bar graphs show the absolute numbers of CD62L^–^CD44^+^ CD4 T cells (**c**), CD62L^–^CD44^+^ CD8 T cells (**d**), or CD62L^+^CD44^+^ CD8 T cells (**e**). Data are representative of 4 independent results. Two-tailed Mann-Whitney test. **f**. CD62L^+^CD44^+^ CD8 T cells were sorted from wild-type or LAT G135D mice (8-week old). Sorted CD62L^+^CD44^+^ CD8 T cells were then stimulated with anti-CD3 antibody (1 μg/ml) overnight at 37^o^C. The expression of CD69 was examined on the next day. Bar graphs summarize the percentage of CD69^+^ T cells. Data are representative of 3 independent experiments. At 0 μg/ml: ns = 0.9807 (bottom left), ns = 0.8325 (bottom right), ns = 0.8784 (middle), ns = 0.9060 (top). At 0.5 μg/ml: P*** = 0.002, P**** < 0.0001, P* = 0.0107 (middle), P* = 0.0221 (top). At 5 μg/ml: ns = 0.7696 (bottom left), ns = 0.9485 (bottom right), ns = 0.9899 (middle), ns = 0.9939 (top). 2-way ANOVA. Data are representative of 4 independent results. **g.h**. Bar graphs summarize the frequency of CD62L^–^CD44^+^ CD4 T cells (**g**), CD62L^–^CD44^+^ CD8 T cells (**h**), or CD62L^+^CD44^+^ CD8 T cells (**h**) from various lymph nodes (as indicated) from 6-week old wild-type or G135D mice. n = 7; P*** = 0.0006. Two-tailed Mann-Whitney test. Data are representative of 3 independent results. **i,j**. Bar graphs summarize the proliferation index as shown in Fig. 3f (in **c**) and 3 h (in **d**). n = 4. Data are representative of 3 experiments. Data are presented as mean values ± SD. **a-d**. Each dot represents an individual mouse. ***P = 0.0005; ****P < 0.0001 (**a**, left); ***P = 0.0003 (**a**, right); ns = 0.5949 (**a**); ns = 0.8381 (**b**, left); ns = 0.0627 (**b**, middle left); ns = 8063 (**b**, middle right); ns = 0.1160 (**b**, right). Two-tailed Mann-Whitney test. Source data

To investigate how T cells with altered kinetic proofreading and potentially enhanced self-reactivity respond in the periphery, we examined the phenotypic and functional characteristics of polyclonal peripheral LAT^G135D^ CD4 and CD8 splenocytes. LAT^G135D^ mice had fewer CD8 T cells (Extended Data Fig. 5a) than their wild-type littermates, whereas LAT^G135D^-expressing CD4 T cells were relatively less affected (Extended Data Fig. 5b). Nonetheless, both LAT^G135D^ CD4 and CD8 populations included enlarged CD62L^–^CD44^+^ populations that were age dependent and obvious only in adult mice (Fig. 3a–d and Extended Data Fig. 5c,d). Interestingly, the LAT^G135D^ mice also harbored a substantial population of CD8 T cells that adopted a central memory-like phenotype (Fig. 3c,e and Extended Data Fig. 5e)—a population that is driven by higher self-reactivity^33,34^ and exhibited enhanced responsiveness to lower-dose anti-CD3 stimulation (Extended Data Fig. 5f). Similar phenotypes were also observed in lymph nodes (Extended Data Fig. 5g,h).

To further investigate the mechanisms behind the altered cellularity in the periphery of LAT^G135D^ mice, we adoptively transferred sorted naive LAT^G135D^ CD4 T cells labeled with CellTrace Violet proliferation dye into sublethally irradiated congenic CD45.1^+^ C57BL/6 hosts to examine the homeostatic proliferation. After 4 days, we observed that LAT^G135D^ CD4 T cells proliferated more robustly than wild-type CD4 T cells (Fig. 3f,g and Extended Data Fig. 5i); approximately 80% of LAT^G135D^ CD4 T cells underwent proliferation compared with 49% of wild-type CD4 T cells (Fig. 3g). Naive LAT^G135D^ CD8 T cells displayed similarly stronger proliferation than wild-type CD8 T cells (Fig. 3h,i and Extended Data Fig. 5j). Notably, transfer into sublethally irradiated MHC-II^–/–^ hosts, which prevents interaction with pMHC-II, rendered LAT^G135D^ CD4 T cells nonproliferative (Fig. 3f,g). Restricting the repertoire of MHC-I-bound self-peptides by employing sublethally irradiated *Tap1*^–/–^*B2m*^–/–^ hosts revealed similar self-pMHC-driven homeostatic proliferation of LAT^G135D^ CD8 T cells (Fig. 3h,i). These data show that LAT^G135D^ T cells exhibit enhanced reactivity/responsiveness to self-pMHCs, which contributes to their greater homeostatic proliferation potential.

### LAT^G135D^ T cells exhibit hyper-responsiveness to self-ligands

**Extended Data Fig. 6:**
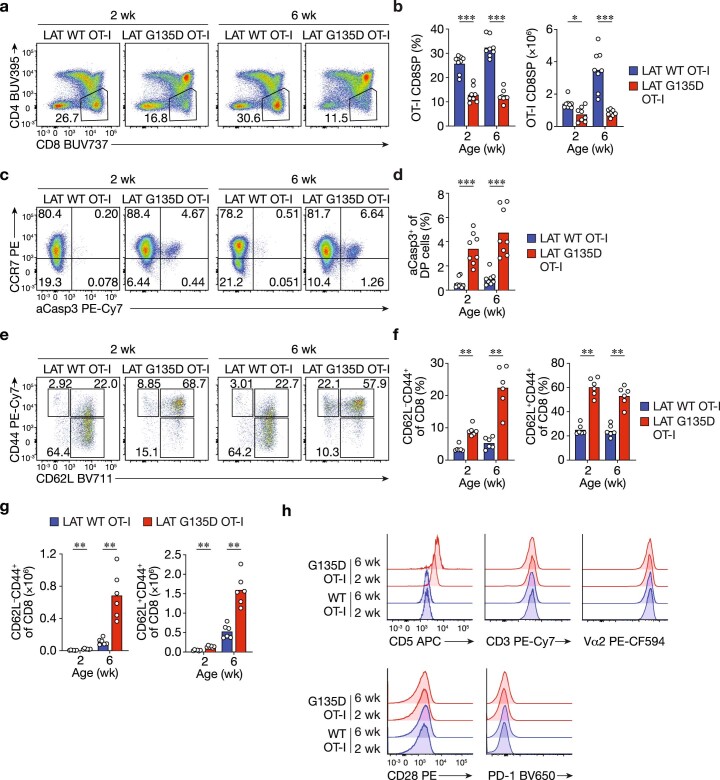
Thymic and peripheral T cell phenotypes in G135D LAT.OT-I.*Rag1*^–/–^ mice recapitulate those in G135D mice on the polyclonal C57BL/6 background. **a**. Pseudocolor flow plots show the expression of CD4 and CD8 on live thymocytes harvested from neonatal or adult wild-type or G135D LAT.OT-I.*Rag1*^–/–^ mice. **b**. Bar graphs show the percentage of CD8 OT-I.*Rag1*^–/–^ T cells in wild-type or G135D LAT mice at the indicated ages. **c,d**. Representative flow plots or bar graphs show the expression of CCR7 and cleaved caspase 3 (aCasp3) in DP thymocytes. **e,f**. Representative pseudocolor plots depict the expression of CD62L and CD44 (**e**). Bar graphs summarize the frequencies of CD62L^–^CD44^+^ (left) and CD62L^+^CD44^+^ (right) CD8 OT-I.*Rag1*^–/–^ T cells. **g**. Bar graphs show the absolute numbers of CD62L^–^CD44^+^ or CD62L^+^CD44^+^ CD8 OT-I.*Rag1*^–/–^ T cells in wild-type or G135D LAT mice at the indicated ages. **h**. Representative histograms display the expression of CD5, CD3, Vα2, CD28 and PD-1 on wild-type or G135D LAT.OT-I.*Rag1*^–/–^ CD8 T cells. **a-g**. Each dot represents an individual mouse; n = 8 (**b**); n = 8 (**d**); n = 5 (**f**); n = 6 (**g**); ***P = 0.0002 (**b**); ***P = 0.0002 (**d**); **P = 0.0022 (**f**); **P = 0.0022 (**g**). Two-tailed Mann-Whitney test. Source data

**Extended Data Fig. 7:**
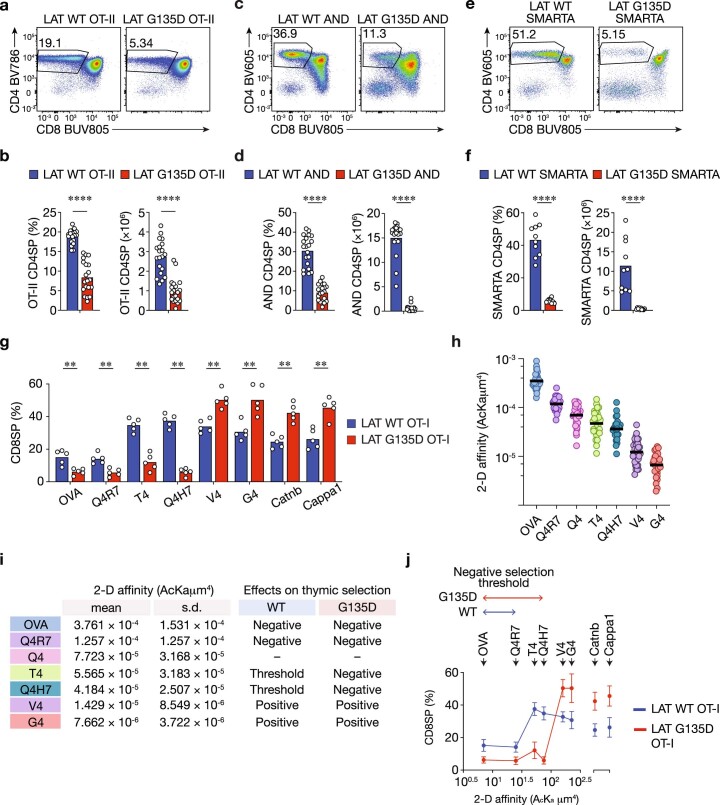
G135D LAT lowers negative selection threshold and enhances productive positive selection. **a–f**. Pseudocolor flow plots show the expression of CD4 and CD8 on live thymocytes harvested from adult wild-type or G135D LAT.OT-II.*Rag1*^–/–^ mice (**a**), LAT.AND.*Rag1*^–/–^ mice (**c**), or LAT.SMARTA.*Rag1*^–/–^ mice (**e**). Bar graphs show the percentage (left) or absolute numbers (right) of CD4SP OT-II.*Rag1*^–/–^ (**b**), LAT.AND.*Rag1*^–/–^ (**d**), or LAT.SMARTA.*Rag1*^–/–^ (**f**) thymocytes in wild-type or G135D LAT mice. P**** = < 0.0001; Two-tailed Mann-Whitney test. Data are representative of 5 independent results. **g**. Bar graphs show the frequency of developed wild-type or G135D LAT.OT-I.*Rag1*^–/–^*Tap1*^–/–^ CD8SP cells in fetal thymic organ culture (FTOC) on day 4. Dots represent individual FTOC culture. Data are compiled from two experiments. n = 5; P** = 0.0079; Two-tailed Mann-Whitney test. **h,i**. Dot plots in (**b**) show the 2-D affinity of OT-I CD8 T cells binding to OVA- or variant-loaded H-2K^b^ (with murine β2 m) coated red blood cells. The mean ± standard deviation are summarized in the table (**c**). Each symbol represents one measurement. n = 33 (OVA, Q4, V4); n = 30 (T4, G4); n = 24 (Q4R7; Q4H7). **j**. Percentage of CD8SP cells developed in the FTOC (as in **g**) versus the 2-D affinity of OT-I TCR for OVA or APLs (as in **h**,**i**). The threshold for negative selection is marked by the horizontal lines on top of the plots. Source data

To more thoroughly study the effects of LAT^G135D^ on self-pMHC reactivity, we introduced the LAT^G135D^ mutation onto the OT-I TCR transgenic *Rag1*^–/–^ background. LAT^G135D^.OT-I.*Rag1*^–/–^ mice exhibited phenotypes consistent with those observed in polyclonal C57BL/6 mice, including a smaller CD8SP population (Extended Data Fig. 6a,b) and enhanced negative selection (Extended Data Fig. 6c,d) in the thymus, as well as augmented CD44^+^ populations in the periphery (Extended Data Fig. 6e–g). CD5 expression was also elevated in LAT^G135D^.OT-I.*Rag1*^–/–^ CD8 T cells, while the expression levels of OT-I TCR (Vα2), CD3 and CD28 were comparable to those of wild-type T cells (Extended Data Fig. 6h). Similar phenotypes resulting from LAT^G135D^ alteration were also observed on OT-II.*Rag1*^–/–^, SMARTA.*Rag1*^–/–^ and AND.*Rag1*^–/–^ TCR transgenic backgrounds (Extended Data Fig. 7a–f).

**Fig. 4:**
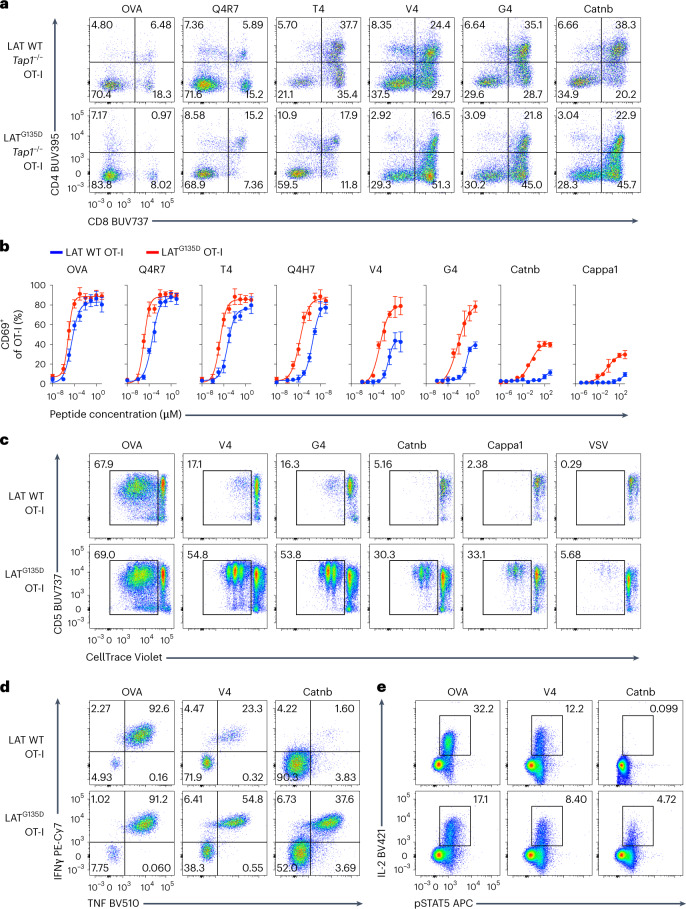
LAT^G135D^ promotes OT-I CD8 T-cell effector function and augments sensitivity to weak ligand stimuli. **a**, Fetal thymi from wild-type or LAT^G135D^.OT-I.*Rag1*^–/–^.*Tap1*^–/–^ mice were cultured with OVA peptide, OVA APLs or self-peptides, as indicated. The percentages of CD8SP cells were analyzed on day 4. The data are representative of two independent experiments. Representative flow plots show the development of CD8SP cells in FTOC. **b**, Naive wild-type or LAT^G135D^.OT-I.*Rag1*^–/–^ TCR transgenic CD8 T cells were sorted from 4- to 5-week-old mice and stimulated overnight with TCRα^–/–^ antigen-presenting cells pulsed with OVA peptide, OVA APLs or self-peptide Catnb or Cappa1 over a wide range of peptide concentrations (as indicated on the *x* axis). The upregulation of CD69 was analyzed the next day by flow cytometry. CD69^+^ cells were plotted against peptide concentrations. The data represent means ± s.d. (*n* = 3 independent experiments). **c**, Naive wild-type or LAT^G135D^.OT-I.*Rag1*^–/–^ TCR transgenic CD8 T cells were sorted from 4- to 5-week-old mice, labeled with CellTrace Violet and cocultured with TCRα^–/–^ antigen-presenting cells pulsed with OVA, APLs (V4 or G4), self-peptide (Catnb or Cappa1) or unrelated peptide (VSV). The fluorescence profile of CellTrace Violet and expression of CD5 were assessed on day 4. The data are representative of at least three independent experiments. **d**, Representative flow plots depicting the production of the cytokines TNF and IFNγ by naive cells from wild-type or LAT^G135D^.OT-I.*Rag1*^–/–^ mice stimulated with OVA-, V4- or Catnb-pulsed TCRα^–/–^ antigen-presenting cells overnight. The data are representative of three independent experiments. **e**, Naive cells from wild-type or LAT^G135D^.OT-I.*Rag1*^–/–^ mice were sorted from 4- to 5-week-old mice and stimulated with OVA-, V4- or Catnb-pulsed TCRα^–/–^ antigen-presenting cells overnight. The production of IL-2 and expression of pSTAT5 were measured by intracellular staining and flow cytometry. The data are representative of four independent experiments. Source data

To further test whether the expression of LAT^G135D^ regulates T-cell ligand discrimination, we utilized four altered peptide ligands (APLs) and two self-peptides, Catnb and Cappa1, that are recognized by the OT-I TCR^35^. Using in vitro fetal thymic organ cultures (FTOCs)^36,37^, we observed that significantly fewer CD8SP cells developed in LAT^G135D^.OT-I.*Rag1*^–/–^.*Tap1*^–/–^ cultures than in wild-type LAT cultures treated with the agonist ovalbumin (OVA), or partial agonists Q4R7 or T4 (Fig. 4a and Extended Data Fig. 7g). In contrast, treatment with the weak agonists V4 and G4 or the self-peptide Catnb promoted stronger positive selection of LAT^G135D^.OT-I.*Rag1*^–/–^.*Tap1*^–/–^ thymocytes, as indicated by a roughly twofold increase in CD8SP cells compared with those in wild-type LAT cell cultures (Fig. 4a and Extended Data Fig. 7g). Further analysis of OVA APL two-dimensional (2D) affinity (Extended Data Fig. 7h,i), along with the frequency of CD8SP, showed that the expression of LAT^G135D^ converts the borderline negative selectors (for example, T4 and Q4H7) into pure negative selectors and augments the selection efficiency of positive selectors (for example, V4, G4, Catnb and Cappa1) (Extended Data Fig. 7j).

**Extended Data Fig. 8:**
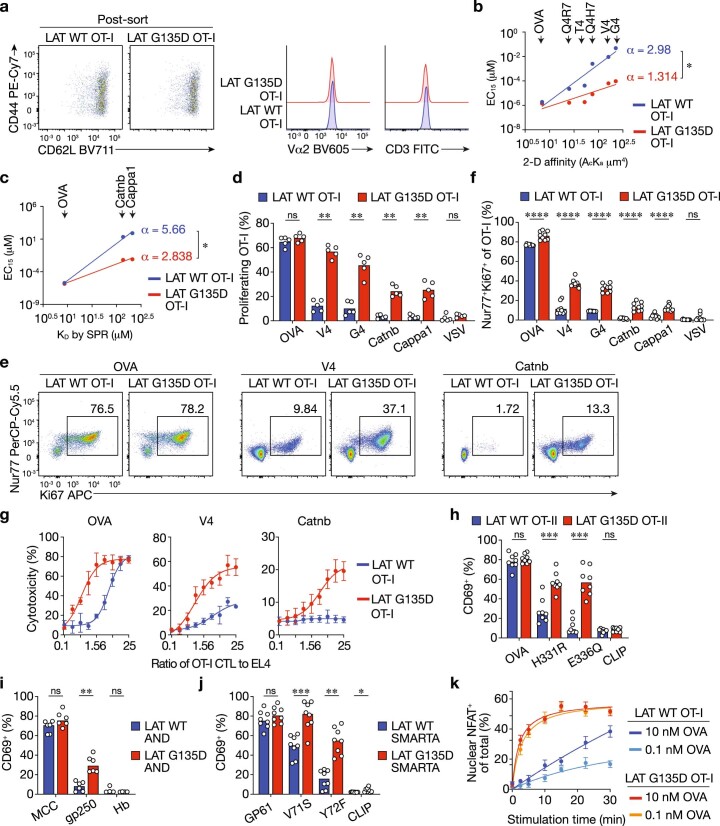
G135D LAT.OT-I.*Rag1*^–/–^ CD8 T cells are ultra-sensitive to very weak ligands and self-peptides. **a**. Representative flow plots of post-sorted naive CD44^–^CD62L^+^ wild-type or G135D CD8 T cells. Histogram plots show the expression level of TCR Vα2 or CD3. **b**. The plot shows the potency (EC_15_ from Fig. 4b) over the 2-D affinity. The 2-D affinities of individual ligands are included in **b**,**c**. Linear regression analysis was performed to obtain the slope (α) as the measure of TCR discriminatory power. The computed α values for wild-type and G135D LAT-expressing T cells are indicated. *P = 0.0200; F = 8.387; DFn = 1; DFd = 8; the two linear regression lines are significantly different. Two-tailed ANCOVA analysis. **c**. The plot depicts the potency (concentration producing 15% maximal effect, or EC_15_, from Fig. 4b) over the published 3-D affinity. Linear regression analysis was performed to obtain the slope (α) as the measure of TCR discriminatory power. The computed values for wild-type or G135D LAT-expressing T cells are indicated. *P = 0.0257; F = 37.38; DFn = 1; DFd = 2; the two linear regression lines are significantly different. Two-tailed ANCOVA analysis. **d**. Bar graphs show the percent of proliferating wild-type LAT.OT-I.*Rag1*^–/–^ or G135D LAT.OT-I.*Rag1*^–/–^ CD8 T cells in response to agonist (OVA), very weak OVA APL (V4 &G4), or self-peptide (Catnb & Cappa1) stimuli. Data are representative of five experiments. **e,f**. Wild-type LAT.OT-I.*Rag1*^–/–^ or G135D LAT.OT-I.*Rag1*^–/–^ CD8 T cells were isolated and stimulated with peptide-pulsed TCRα^–/–^ antigen-presenting cells overnight. The expression of Ki67 and endogenous Nur77 (not the eGFP reporter) was analyzed by intracellular staining the next day. Representative pseudocolor plots (**b**) and bar graphs (**c**) are shown. Data are representative of three independent experiments. **g**. Cytotoxicity assays demonstrating wild-type or G135D LAT cytotoxic T lymphocyte (CTL)-mediated killing of EL4 cells pulsed with 1 μM OVA, V4 peptide, or self-peptide Catnb. EL4 cells were mixed with CTL at the indicated ratios. Killing capacities were assessed after 4 hours. Data are representative of four independent experiments. **h–j**. CD69 upregulation of sorted naive LAT.OT-II.*Rag1*^–/–^ (h), LAT.AND*.Rag1*^–/–^ mice (i), or LAT.SMARTA.*Rag1*^–/–^ (j) CD4 T cells in responded to 1 μM of peptide-pulsed antigen-presenting cells (as indicated). OVA is an agonist for OT-II TCR, whereas H331R and E336Q are two weaker its altered peptide ligands. MCC is an agonist for AND TCR, whereas gp250 is an identified positively selecting self-peptide for AND TCR. GP61 is an agonist for SMARTA TCR, whereas V71S and Y72F are weaker altered peptide ligands for SMARTA TCR. CLIP or Hb peptides are used as negative controls. Data are representative of 5 independent experiments for h, 3 independent experiments for i, and 3 independent experiments for j. **k**. The first 30 min of NFAT nuclear translocation kinetics in wild-type LAT.OT-I.*Rag1*^–/–^ or G135D LAT.OT-I.*Rag1*^–/–^ CD8 T cells in response to various doses of OVA-pulsed TCR Cα^–/–^ antigen-presenting cells, as shown in Fig. 5a. **d,f,h,i,j**. Each dot represents a sample from an individual mouse. **P = 0.0079; ****P < 0.0001; ns = 0.0222 (**d**); ns = 0.5021 (**f**). ***P = 0.0005 (left); ***P = 0.0002 (right); ns = 0.5737 (left); ns = 0.2345 (right) (**h**); **P = 0.0022; ns = 0.1320 (left); ns = 0.8857 (right) (**i**); ns = 0.3423; ***P = 0.0002; **P = 0.0011; *P = 0.0049 (**j**); Two-tailed Mann-Whitney test. Source data

Next, we isolated naive LAT^G135D^ or wild-type LAT.OT-I.*Rag1*^–/–^ peripheral CD8 T cells from 4- to 5-week-old mice (Extended Data Fig. 8a), stimulated the cells with OVA- or APL-pulsed antigen-presenting cells and examined the upregulation of CD69 (Fig. 4b). Whereas LAT^G135D^.OT-I.*Rag1*^–/–^ CD8 T cells responded only slightly more sensitively than wild-type OT-I.*Rag1*^–/–^ CD8 T cells to OVA or the partial agonists Q4R7, T4 and Q4H7, they responded with substantially greater sensitivity to the weak ligands V4 and G4 and self-peptides Catnb and Cappa1 (Fig. 4b). Plotting the potency by 2D (Extended Data Figs. 8b) or 3D (Extended Data Fig. 8c) affinity revealed that the expression of LAT^G135D^ lowers the TCR discriminatory power (flattening the slope on the log–log plot)^4^, particularly in response to the weak ligands and self-peptides.

These weak ligands or self-peptides also promoted robust proliferative responses by naive LAT^G135D^.OT-I.*Rag1*^–/–^ CD8 T cells in contrast with wild-type cells, as revealed by the dilution of CellTrace Violet dye (Fig. 4c and Extended Data Fig. 8d). Similarly, after culture with OVA- or APL-pulsed antigen-presenting cells, a significantly greater number of LAT^G135D^ compared with wild-type LAT.OT-I.*Rag1*^–/–^ CD8 T cells were Ki-67^+^ cells the next day (Extended Data Fig. 8e,f). These Ki-67^+^ cells also exhibited upregulation of endogenous Nur77 (Extended Data Fig. 8f), which is evidence for TCR recognition-driven proliferation. In addition, the weak ligand V4 and self-peptide Catnb induced more LAT^G135D^ versus wild-type LAT.OT-I.*Rag1*^–/–^ CD8 T cells to produce the cytokines interferon-γ (IFNγ) and tumor necrosis factor (TNF) (Fig. 4d). Interestingly, the expression of LAT^G135D^ had the opposite effect on the production of interleukin-2 (IL-2) (Fig. 4e). Next, we generated cytotoxic T lymphocytes (CTLs) and found that LAT^G135D^.OT-I.*Rag1*^–/–^ CTLs mediated greater cytotoxicity against APL-pulsed EL4 cells at lower CTL-to-EL4 ratios than wild-type LAT.OT-I.*Rag1*^–/–^ CTLs (Extended Data Fig. 8g). Weaker ligands or self-peptides were also able to activate LAT^G135D^ CD4 T cells expressing OT-II, SMARTA or AND TCRs (Extended Data Fig. 8h–j) to a greater degree than wild-type LAT CD4 T cells. These results suggest that the expression of LAT^G135D^ may enable T cells to adopt a stronger effector cell program when challenged with weaker pMHCs or even self-pMHCs, suggesting that LAT^G135D^ OT-I cells are less able to discriminate a true agonist from a weak agonist or even a self-pMHC.

### LAT^G135D^ facilitates the nuclear translocation of NFAT

**Fig. 5:**
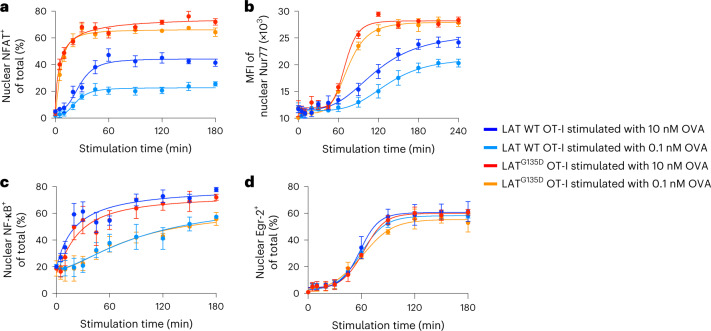
LAT^G135D^-mediated signaling promotes NFAT1 and Nur77 translocation into the nucleus. **a**–**d**, Naive wild-type or LAT^G135D^.OT-I.*Rag1*^–/–^ CD8 T cells from 4- to 5-week-old mice were sorted and subjected to nuclear staining with CellTrace Blue dyes, then stimulated in vitro with 10 or 0.1 nM OVA peptide-pulsed TCRα^–/–^ splenocytes over a time course of 180 or 240 min (as indicated on *x* axis). Cell nuclei were isolated according to a published protocol, fixed and permeabilized, then subjected to antibody staining for NFAT1, Nur77, NF-κB or Egr-2. Nuclear NFAT1 (**a**), Nur77 (**b**), NF-κB (**c**) and Egr-2 (**d**) expression was analyzed by flow cytometry. The percentage of positive nuclei (for NFAT1, NF-κB and Egr-2) or mean fluorescence intensity (MFI; Nur77) for individual conditions was plotted against the stimulation time to depict the nuclear translocation kinetics of transcription factors, as indicated. The data represent means ± s.d. (*n* = 4 independent experiments). Source data

To determine how the altered LAT Y136-centric kinetic proofreading step modulates the activation of specific transcription factors that are responsive to distinct signaling pathways, we examined the activation of transcription factors in isolated cell nuclei^38^ from naive wild-type or LAT^G135D^.OT-I.*Rag1*^–/–^ CD8 T cells (Fig. 5). We first characterized nuclear NFAT1, which translocates from the cytoplasm to the nucleus following its dephosphorylation by the calcium–calmodulin-activated phosphatase calcineurin, the consequence of direct LAT–PLC-γ1–calcium downstream signaling. OVA stimulation induced rapid nuclear localization of NFAT1, and the expression of LAT^G135D^ substantially promoted increased accumulation of NFAT1 in nuclei (Fig. 5a and Extended Data Fig. 8k). At none of the responses of the wild-type cells did NFAT translocation equal that of the LAT^G135D^ variant. NFAT signaling is necessary for the induction of transcripts of Nur77 (ref. ^39^), and the magnitude of the nuclear expression of Nur77 was also greatly enhanced in LAT^G135D^.OT-I.*Rag1*^–/–^ CD8 T cells (Fig. 5b). Interestingly, we did not observe substantial differences in nuclear translocation of nuclear factor-κB (NF-κB) (Fig. 5c) or Egr-2 (Fig. 5d) between LAT^G135D^ and wild-type LAT.OT-I CD8 T cells, which are regulated through costimulatory signaling in addition to TCR signals^40,41^. These data suggest that LAT^G135D^-promoted PLC-γ1 and calcium signals enhance the nuclear translocation of NFAT1 and transcriptional induction of Nur77, which is highly sensitive to NFAT, both of which may contribute to the hyper-responsiveness of LAT^G135D^ T cells.

### G135D LAT augments T-cell expansion to *Listeria* in vivo

**Fig. 6:**
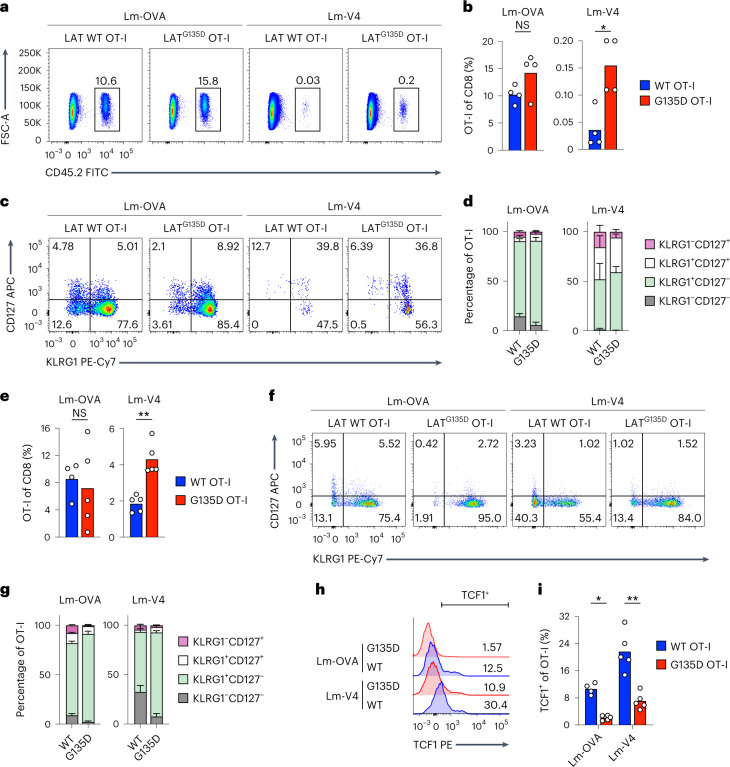
LAT^G135D^ augments the CD8 T-cell response in vivo. **a**, Representative pseudocolor plots showing the frequency of CD45.2 OT-I T cells among total CD8 T cells on day 7 postinfection of *L. monocytogenes* expressing OVA (Lm-OVA) or V4 (Lm-V4). **b**, Bar graphs depicting the frequency of OT-I T cells among total CD8 T cells on day 7 postinfection (*n* = 4). *P = 0.0286 and NS = 0.2. **c**, Representative pseudocolor plots of the expression of KLRG1 and CD127 on wild-type or LAT^G135D^.OT-I.*Rag1*^–/–^ CD8 T cells on day 7 postinfection. **d**, Bar graphs summarizing the relative distribution of each subset based on the expression of KLRG1 and CD127 (as in **c**) of wild-type or LAT^G135D^.OT-I.*Rag1*^–/–^ CD8 T cells on day 7 postinfection (*n* = 4). **e**, Bar graphs summarizing the percentage of OT-I T cells among total spleen CD8 T cells 4 days after rechallenge with VSV-OVA (*n* = 4 for wild-type donor and Lm-OVA first infection, *n* = 5 for LAT^G135D^ donor and Lm-OVA infection, as well as wild-type or LAT^G135D^ donor and Lm-V4 first infection). **P = 0.0079 and NS = 0.6863. **f**, Representative pseudocolor plots of the expression of KLRG1 and CD127 on wild-type or LAT^G135D^.OT-I.*Rag1*^–/–^ CD8 T cells on day 7 after VSV-OVA rechallenge. **g**, Bar graphs depicting the frequency of each subset based on the expression of KLRG1 and CD127 on OT-I T cells in the spleen 4 days after rechallenge with VSV-OVA (*n* = 4 for wild-type donor and Lm-OVA first infection, *n* = 5 for LAT^G135D^ donor and Lm-OVA infection, as well as wild-type or LAT^G135D^ donor and Lm-V4 first infection). **h**, Representative histograms of the expression of TCF1 in wild-type or LAT^G135D^.OT-I.*Rag1*^–/–^ CD8 T cells analyzed on day 4 after VSV-OVA rechallenge. **i**, Bar graph quantifying the percentage of TCF1^+^ cells (the positive horizontal bar gate shown in **h**) (*n* = 4 for wild-type donor and Lm-OVA first infection and *n* = 5 for LAT^G135D^ donor and Lm-OVA infection, as well as wild-type or LAT^G135D^ donor and Lm-V4 first infection). *P = 0.0159 and **P = 0.0079. In **a**–**i**, the data are representative of at least three independent experiments. The dots in **b**, **e** and **i** represent individual mice. Statistical significance was determined by two-tailed Mann–Whitney *U*-test. In **d** and **g**, the data represent means ± s.d. Source data

**Extended Data Fig. 9:**
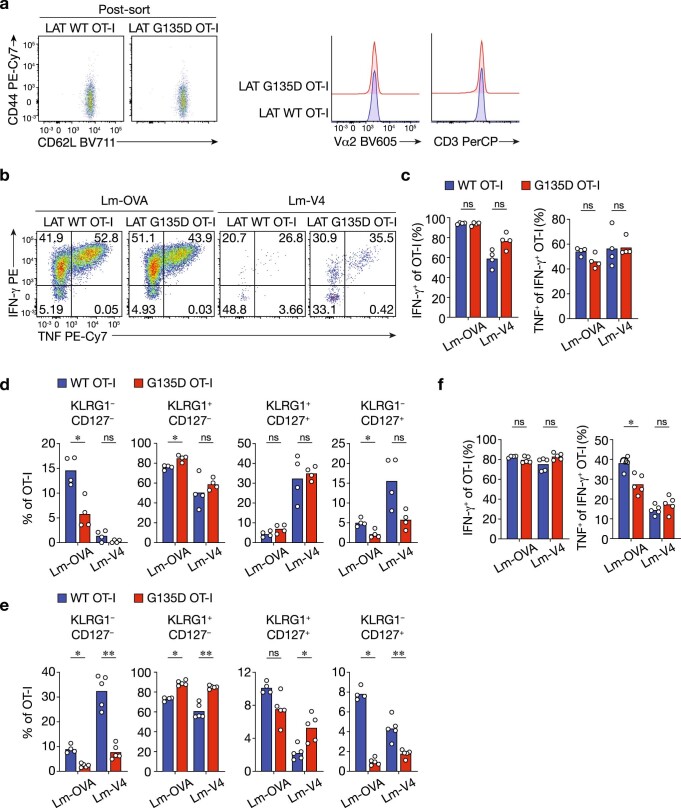
G135D LAT.OT-I.*Rag1*^–/–^ CD8 T cells exhibit proliferation fitness and skewing toward terminal differentiation during recombinant *Listeria* infection. **a**. Representative flow plots of post-sorted naive CD44^–^CD62L^+^ wild-type or G135D OT-I CD8 T cells. **b**. Splenocytes collected from day 7 post-infection were restimulated with corresponding antigens OVA or V4, and the production of cytokines was assessed by intracellular staining and analyzed by flow cytometry. The representative pseudocolor plots depict the production of IFN-γ and TNF after restimulation. The number in each quadrant represents the percentage of OT-I CD8 T cells producing the indicated cytokine. **c**. Bar graphs summarize the percentage of wild-type or G135D LAT.OT-I.*Rag1*^-/-^ CD8 T cells producing IFN-γ (left) or TNF^+^ of IFN-γ + OT-I cells (right). Dots represents single mice; n = 4. ns = 0.9714 (left); ns = 0.0571 (middle left); ns = 0.0571 (middle right); ns = 0.8857 (right). Two-tailed Mann-Whitney test. **d**,**e**. Bar graphs show the frequency of the KLRG1^–^CD127^–^,KLRG1^+^CD127^–^, KLRG1^+^CD127^+^, and KLRG1^–^CD127^+^ subsets of OT-I.*Rag1*^–/–^ or G135D LAT.OT-I.*Rag1*^–/–^ CD8 T cells on day 7 post-infection (**d**) or on day 4 after rechallenge with VSV-OVA (**e**). Each dot represents one individual mouse; n = 4 (**c**); n = 4 (WT donor cells primed with Lm-OVA, **e**) or n = 5 (all other conditions, **e**). *P = 0.0286 (**d**); *P = 0.0159 (**e**); ns = 0.2286 (far left; **d**); ns = 0.3429 (middle left; **d**); ns = 0.1143 (middle; **d**); ns: *P* > 0.9999 (middle right; **d**); ns = 0.0571 (far right; **d**); ns = 0.0635 (**e**). Two-tailed Mann-Whitney test. **f**. Splenocytes collected from day 4 post-infection were restimulated with OVA. The production of cytokines was assessed by intracellular staining and analyzed by flow cytometry. Bar graphs summarize the percentage of wild-type or G135D LAT.OT-I.*Rag1*^–/–^ CD8 T cells producing IFN-γ (left) or TNF^+^ of IFN-γ^+^ OT-I cells (right). Dots represent single mice; n = 4 (WT donor, Lm-OVA first infection); n = 5 (G135D donor, Lm-OVA infection; WT or G135D donor, Lm-V4 first infection). *P = 0.0317; ns = 0.2540 (left); ns = 0.0952 (middle left); ns = 0.3095 (right). Two-tailed Mann-Whitney test. Source data

To examine how these LAT^G135D^ T cells balance tolerance and immune responsiveness, we used an immune challenge model. We adoptively transferred sorted CD62L^+^CD44^–^ naive CD45.2^+^ LAT^G135D^ or wild-type LAT.OT-I.*Rag1*^–/–^ spleen CD8 T cells into congenic CD45.1^+^ hosts (Extended Data Fig. 9a) and infected the mice with recombinant *Listeria monocytogenes* strains engineered to express OVA (Lm-OVA) or very weak APL V4 (Lm-V4) the next day^35^. On day 7 postinfection, LAT^G135D^.OT-I.*Rag1*^–/–^ CD8 T cells consistently expanded to a greater degree than wild-type OT-I*.Rag1*^–/–^ CD8 T cells in the Lm-V4 infection settings (Fig. 6a,b). Notably, OT-I TCR affinity to the V4 peptide is reported to be substantially weaker, within the range of characterized positively selecting APLs^5,35^. Lm-V4 infection resulted in the activation of only ~0.03% of wild-type T cells, but led to expansion of 0.15% of LAT^G135D^ T cells (Fig. 6b). Interestingly, OT-I T cells expressing LAT^G135D^ and wild-type OT-I T cells responded comparably to Lm-OVA, and activated LAT^G135D^.OT-I.*Rag1*^–/–^ CD8 T cells showed comparable cytokine production capacity to their wild-type counterparts (Extended Data Fig. 9b,c). These results are consistent with our in vitro data showing that modification of a kinetic proofreading step has a greater effect on weak ligand stimulation. In addition, infection with Lm-OVA emphasized the shift in effector versus memory cell fate decisions. We observed that the KLRG1^–^CD127^+^ memory precursor population was substantially decreased by more than twofold among transferred LAT^G135D^.OT-I*.Rag1*^–/–^ CD8 T cells compared with transferred LAT wild-type cells in response to Lm-OVA infection (Fig. 6c,d and Extended Data Fig. 9d), whereas the short-lived KLRG1^+^CD127^–^ effector cell population was consistently larger.

Next, we investigated whether the enhanced proliferation of LAT^G135D^.OT-I.*Rag1*^–/–^ CD8 T cells was retained during recall responses. During rechallenge responses with vesicular stomatitis virus expressing OVA (VSV-OVA), LAT^G135D^.OT-I*.Rag1*^–/–^ T cells that had been primed with Lm-V4 maintained their expansion advantage (Fig. 6e). The skewed differentiation of KLRG1^–^CD127^+^ versus KLRG1^+^CD127^–^ cells was even more obvious upon rechallenge (Fig. 6f,g and Extended Data Fig. 9e). After rechallenge, when Lm-OVA-primed OT-I T cells were restimulated in vitro with the agonist OVA, LAT^G135D^.OT-I.*Rag1*^–/–^ cells had inferior TNF production (Extended Data Fig. 9f)—a possible characteristic of terminally differentiated effector cells. Indeed, the T-cell factor-1-positive (TCF1^+^) population of LAT^G135D^.OT-I*.Rag1*^–/–^ T cells was also significantly smaller (Fig. 6h,i). These data suggest that the expression of LAT^G135D^ augments sensitization of T cells to weak ligand stimuli in vivo and modulates cell fate decisions during immune responses.

### LAT^G135D^ female mice show signs of autoimmune pathology

**Fig. 7:**
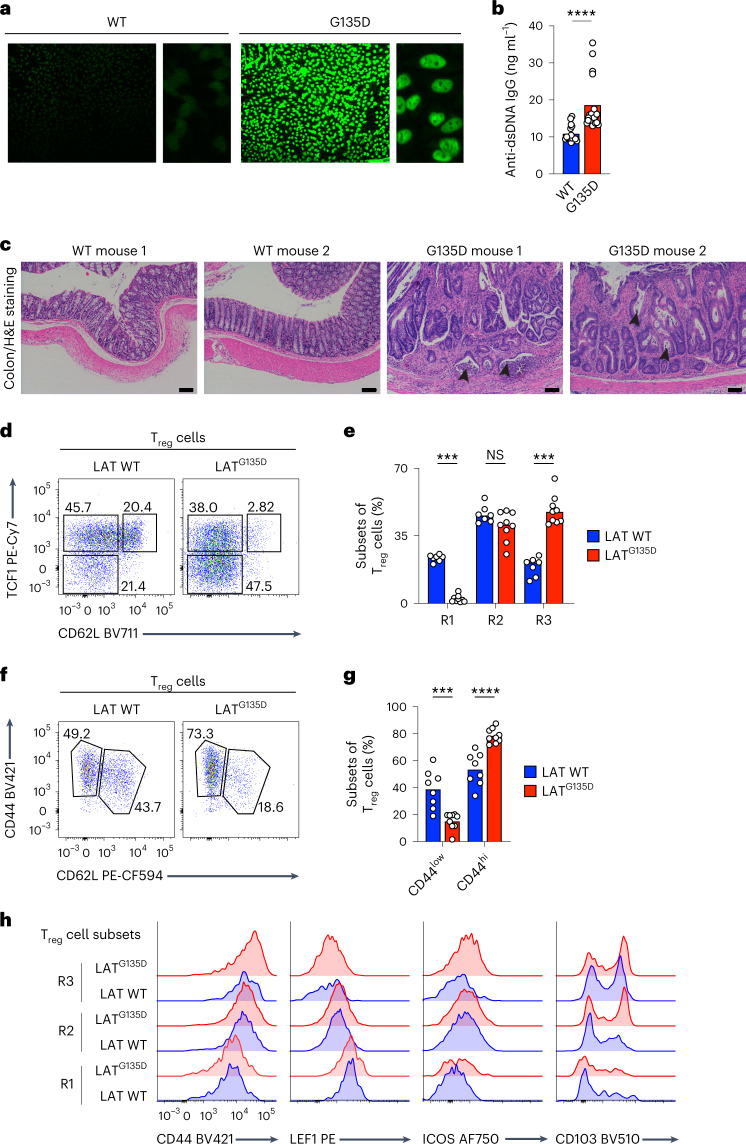
Aged female LAT^G135D^ mice develop higher titers of anti-dsDNA IgG than wild-type counterparts, along with signs of colitis. **a**,**b**, Sera from aged wild-type or LAT^G135D^ female mice (1 year old) were collected and subjected to antinuclear antibody staining (**a**) and anti-dsDNA IgG titers were measured by ELISA (**b**) (*n* = 15 for the wild type and *n* = 24 for LAT^G135D^). ****P < 0.0001. The data are representative of two independent experiments. **c**, Histopathological analysis and hematoxylin and eosin (H&E) staining revealed signs of acute and chronic colitis, including abnormal neutrophil infiltration and crypt destruction/distortion (arrowheads), in aged LAT^G135D^ female mice that were absent from wild-type littermates. The data are representative of two independent experiments. Scale bars, 100 μm. **d**, Representative flow cytometry plots of the expression of CD62L and TCF1 on wild-type and LAT^G135D^ CD25^+^Foxp3^+^ regulatory T cells from 1-year-old mice. **e**, Bar graph summarizing the frequency of each subset as a proportion of total regulatory T cells. The regulatory T-cell subsets R1, R2 and R3 represent CD62L^+^TCF1^+^, CD62L^–^TCF1^+^ and CD62L^–^TCF1^–^ cells, respectively. ***P = 0.0006 (left), ***P = 0.0002 (right) and NS = 0.1142. The data are representative of three independent experiments. **f**, Representative flow cytometry plots of the expression of CD62L and CD44 on wild-type and LAT^G135D^ CD25^+^Foxp3^+^ regulatory T cells from 1-year-old mice. **g**, Bar graph summarizing the frequency of CD44^hi^ and CD44^low^ regulatory T-cell populations as a proportion of total regulatory T cells. ***P = 0.0010 and ****P < 0.0001. The data are representative of three independent experiments. **h**, Expression of CD44, LEF1, ICOS and CD103 of wild-type and LAT^G135D^ CD25^+^Foxp3^+^ regulatory T cells from 1-year-old mice. In **b**, **e** and **g**, statistical significance was determined by two-tailed Mann–Whitney *U*-test. Source data

To determine the effects of altered kinetic proofreading in older mice, we performed serological and histological analyses. Compared with aged wild-type littermate female mice from the same cohort, LAT^G135D^ female mice from two cohort groups demonstrated nuclear staining for autoantibodies in indirect immunofluorescence assays (Fig. 7a). LAT^G135D^ female mice also had higher titers of anti-double-stranded DNA (anti-dsDNA) antibodies in their sera by enzyme-linked immunosorbent assay (ELISA) (Fig. 7b). However, histological examination of hematoxylin and eosin staining of the kidney revealed no significant abnormalities across all samples examined. Further histological examination of the colons of aged female mice revealed extensive cell infiltration in LAT^G135D^ female mice, indicative of severe cryptitis and crypt abscesses; no similar signs of cell infiltration or inflammation were observed in the colons of wild-type female littermates (Fig. 7c). Interestingly, at 1 year of age, regulatory T cells in LAT^G135D^ mice also exhibited enlarged TCF1^–^CD62L^–^ populations (Fig. 7d,e) and upregulated expression of CD44 and other phenotypic markers that are associated with effector regulatory T cells (Fig. 7f–h). Thus, these findings suggest that disruption of proper discrimination via perturbation of LAT Y136 phosphorylation results in hyper-responsiveness to self-ligands and the loss of proper maintenance of long-term tissue homeostasis, particularly at the barrier tissues.

### LAT^G135D^ T cells adapt in the periphery to maintain tolerance

**Fig. 8:**
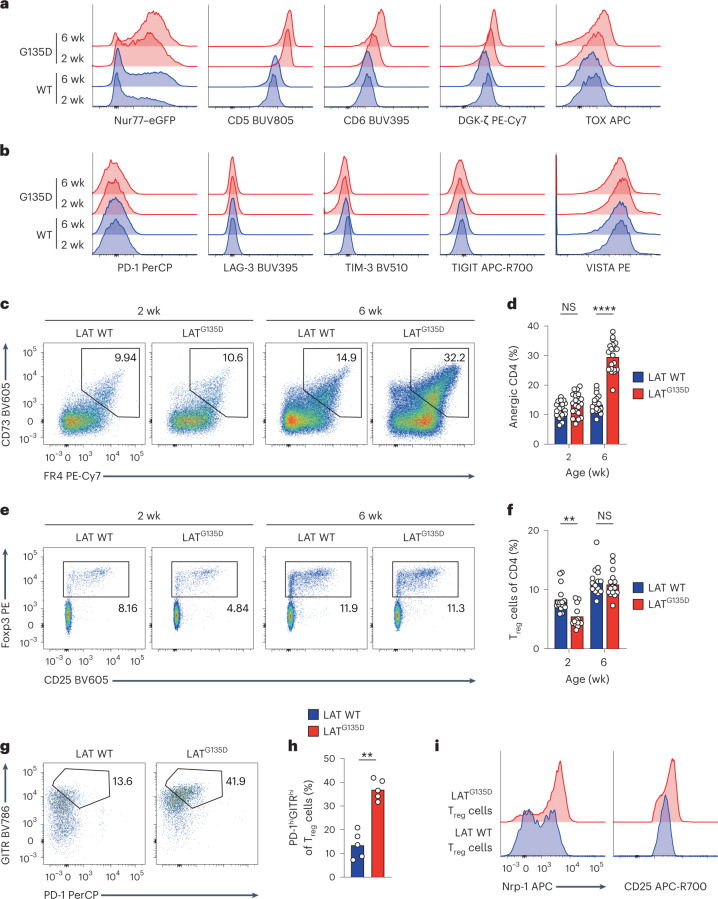
LAT^G135D^ peripheral T cells adapt in an age-dependent manner to maintain tolerance. **a**, Representative histograms of the expression of Nur77–eGFP, CD5, CD6, DGK-ζ and TOX in peripheral CD4 T cells from wild-type and LAT^G135D^ mice (ages as indicated). The data are representative of at least three independent experiments. **b**, Representative histograms of the expression of coinhibitory receptors, including PD-1, LAG-3, TIM-3, TIGIT and VISTA on naive peripheral CD4 T cells from wild-type and LAT^G135D^ mice (ages as indicated). The data are representative of at least three independent experiments. **c**, Representative pseudocolor plots showing the expression of CD73 and FR4 on peripheral Foxp3^–^ CD4 T cells from wild-type and LAT^G135D^ mice (ages as indicated). **d**, Bar graph summarizing the percentages of CD73^hi^FR4^hi^ cells. Each dot represents one mouse (*n* = 20). The data are representative of and compiled from at least five independent experiments. ****P < 0.0001 and NS = 0.0950. **e**, Representative pseudocolor plots showing the expression of Foxp3 and CD25 in peripheral CD4 T cells from wild-type and LAT^G135D^ mice (ages as indicated). **f**, Bar graph summarizing the percentages of Foxp3^+^CD25^+^ cells. Each dot represents one mouse (*n* = 15). The data are representative of and compiled from at least five independent experiments. **P = 0.0037 and NS = 0.4423. **g**, Representative pseudocolor plot showing the expression of PD-1 and GITR in peripheral Foxp3^+^CD25^+^ regulatory T cells isolated from the spleens of 6-week-old wild-type and LAT^G135D^ mice. **h**, Bar graph summarizing the percentage of PD-1^hi^GITR^hi^ as a proportion of total T_reg_ cells. The data are representative of at least five independent experiments. **i**, Histogram showing the expression of Nrp-1 and CD25. The data are representative of at least five independent experiments. In **d**, **f** and **h**, statistical significance was determined by two-tailed Mann–Whitney *U*-test. Source data

**Extended Data Fig. 10:**
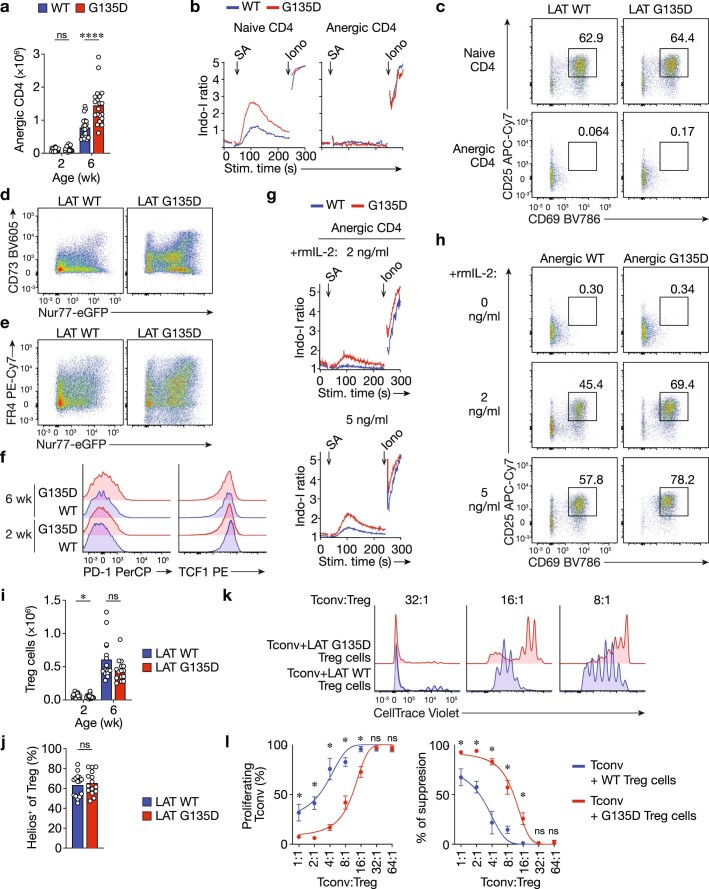
Enlarged G135D anergic T cell populations are functionally hyporesponsive, but IL-2 treatment can restore their function. **a**. Bar graphs show the absolute number of CD73^+^FR4^+^ CD4 T cells in the periphery at different ages. Data are representative of at least five experiments. Each dot represents one mouse. n = 20. ****P < 0.0001; ns = 0.1894. Two-tailed Mann-Whitney test. wk: weeks. **b**. Wild-type or G135D LAT CD4 T cells were isolated and stained with antibodies against CD62L, CD44, CD73, and FR4, and then loaded with calcium dye Indo-I and labeled with biotinylated anti-CD3. Wild-type and G135D CD4 T cells were barcoded with different titrations of CellTrace Violet and pooled together, allowing simultaneous analysis of the cells’ calcium responses upon anti-CD3 crosslinking. Ionomycin treatment served as a positive control. Calcium traces recorded over 5 min are shown. Data are representative of three independent experiments. **c**. Naive or anergic wild-type or G135D LAT CD4 T cells were sorted, and stimulated with plate-bound anti-CD3 and soluble anti-CD28 monoclonal antibodies overnight. The upregulation of CD69 and CD25 was analyzed the next day. Representative pseudocolor contour plots are shown. Data are representative of three independent experiments. **d,e**. Representative flow cytometry plots show the expression of Nur77-eGFP and CD73 (**d**) or FR4 (**e**). Data are representative of at least five independent experiments. **f**. Representative histograms depict the expression levels of PD-1 and TCF1 in CD73^+^FR4^+^Foxp3^–^ anergic CD4 T cells isolated from wild-type or G135D LAT knock-in mice at 2 or 6 weeks of age. Data are representative of two independent experiments. **g**. CD73^+^FR4^+^Foxp3^–^ anergic wild-type or G135D LAT CD4 T cells were sorted and treated with 2 ng/ml or 5 ng/ml recombinant murine IL-2 (rmIL-2; concentration as indicated) overnight and then analyzed for calcium responses. Anergic T cells were labeled with biotinylated anti-CD3 and loaded with calcium dye Indo-I. Calcium responses to anti-CD3 crosslinking were analyzed by flow cytometry for 5 min. Representative calcium traces are shown. Data are representative of three independent experiments. **h**. CD73^+^FR4^+^Foxp3^–^ anergic wild-type or G135D LAT CD4 T cells were sorted and stimulated with plate-bound anti-CD3 and soluble anti-CD28 overnight along with the addition of 2 ng/ml or 5 ng/ml rmIL-2 (concentration as indicated). The upregulation of CD69 and CD25 were analyzed the next day. Representative flow pseudocolor plots are shown. Data are representative of three independent experiments. **i**. Bar graphs show the absolute number of CD25^+^Foxp3^+^ CD4 T cells in the periphery at different ages. Data are representative of at least five experiments. Each dot represents one mouse. n = 15. *P = 0.0164; ns = 0.1261. Two-tailed Mann-Whitney test. **j**. Bar graphs show the percentages of Helios^+^ cells among CD25^+^Foxp3^+^ CD4 T cells in adult wild-type and G135D mice. Data are representative of at least five experiments. Each dot represents one mouse. n = 15. ns = 0.6312. Two-tailed Mann-Whitney test. **k,l**. Wild-type or G135D regulatory T (Treg) cells were sorted from wild-type (WT) or G135D Foxp3-RFP^+^ mice. Polyclonal naive CD8 T cells (Tconv) from CD45.1^+^ C57BL/J mice were purified and labeled with CellTrace Violet dyes and used as responsive cells. CellTrace Violet-labled CD45.1^+^ Tconv cells were co-cultured with titrated ratios of regulatory T cells as indicated. Inhibition of Tconv cell proliferation was used as a readout for Treg cell suppressive function. Representative histograms of Tconv cell proliferation are shown in **k**. Bar graphs in **l** summarize the proliferation of Tconv cells and the suppressive activity of Treg cells. *P = 0.0286; ns = 0.8857. Two-tailed Mann-Whitney test. Data are representative of five independent experiments. Data are presented as mean values ± SD. Source data

LAT^G135D^-induced hyper-responsiveness did not result in spontaneous autoimmune disease in young adult mice. We wondered whether possible compensatory or adaptive mechanisms in the periphery prevented the autoimmune or autoinflammatory phenotypes we observed in older mice. Indeed, in the steady state, LAT^G135D^ CD4 T cells expressed higher levels of key negative regulators of TCR-dependent T-cell responses, including Nur77, CD5, CD6, DGK-ζ and TOX (Fig. 8a). The expression levels of several well-known coinhibitory receptors were surprisingly unaffected in LAT^G135D^ CD4 T cells, including PD-1, LAG-3, Tim-3, TIGIT and VISTA (Fig. 8b). LAT^G135D^ CD4 T cells also developed an age-dependent anergy phenotype, as evidenced by an increase in Foxp3^–^CD73^+^FR4^+^ CD4 T cells in frequency (Fig. 8c,d) and in absolute number (Extended Data Fig. 10a) as mice aged from 2–6 weeks postnatally. These anergic CD4 cells failed to induce calcium increases in response to anti-CD3ε and anti-CD28 stimulation (Extended Data Fig. 10b) and did not upregulate CD25 or CD69 (Extended Data Fig. 10c), revealing their hyporesponsiveness. In addition, in LAT^G135D^.Nur77–eGFP reporter mice, the CD73^hi^ (Extended Data Fig. 10d) or FR4^hi^ cells (Extended Data Fig. 10e) were predominantly enriched in the Nur77–eGFP^hi^ population, suggesting that continued TCR self-pMHC stimulation may have driven the emergence of the population. The CD73^+^FR4^+^ LAT^G135D^ CD4 T-cell population retained low expression of the activation marker PD-1 and high expression of the stemness regulator TCF1 (Extended Data Fig. 10f), consistent with clonal anergy rather than exhaustion^42–44^. IL-2 treatment^42,44^ of the sorted CD73^+^FR4^+^ wild-type or LAT^G135D^ CD4 T cells at least partially reversed their unresponsive state (Extended Data Fig. 10g,h), and the formerly anergic LAT^G135D^ T cells still mounted stronger responses than the formerly anergic wild-type T cells. Surprisingly, the frequency and size of the T_reg_ cell population did not change significantly between wild-type and LAT^G135D^ mice as they aged (Fig. 8e,f; absolute number in Extended Data Fig. 10i), nor did the expression of the transcription factor Helios vary between LAT^G135D^ and wild-type T_reg_ cells (Extended Data Fig. 10j). Interestingly, despite this, LAT^G135D^ regulatory T cells displayed higher expression of PD-1, GITR, CD25 and Nrp-1 (Fig. 8g–i), which are markers reported to associate with superior suppressive function. Indeed, LAT^G135D^ regulatory T cells exhibited stronger suppressive activities than wild-type regulatory T cells when cocultured with CellTrace Violet-labeled conventional CD8 T cells (Extended Data Fig. 10k,l). Taken together, our data suggest that LAT^G135D^ augments self-pMHC sensitivity and may trigger intrinsic adaptive mechanisms to maintain peripheral tolerance (Supplementary Fig. 1).

## Discussion

A kinetic proofreading model was developed to explain the remarkable discriminatory power of TCR ligand recognition—a process central to T-cell fate decisions during development and immune responses^4,7,11^. However, until now, a lack of animal models allowing manipulation of a bona fide proofreading step has hampered our understanding of the importance of safeguarding TCR ligand discrimination in physiological and pathological settings. Here we generated a robust in vivo mouse model, harboring the LAT^G135D^ alteration, in which T cells are hardwired to shorten the time delay for TCR–pMHC input signals to trigger activation. We showed that shortening the time of molecular engagement required for a key step in kinetic proofreading allows antigens with low signal strength that normally fail to generate effective T-cell responses to serve as activating signals. LAT^G135D^-expressing T cells engage robust central and peripheral tolerance and display heightened effector responses to pathogens. However, LAT^G135D^-mediated alterations also impair the formation of memory precursors and predispose female mice to features associated with autoimmunity. Thus, our findings suggest that the slow rate of LAT Y136 phosphorylation establishes a level of proper TCR ligand discrimination that allows T cells to scale responses accordingly to distinguish between ligands spanning a broad range of potencies and affinities. Our results emphasize the importance of slow phosphorylation of LAT Y136 to maintain T-cell unresponsiveness toward self-peptides and, therefore, tolerance.

Editing to shorten the signaling delay after TCR:pMHC engagement revealed the importance of the evolutionarily conserved slow kinetics of the LAT Y136 proofreading step. The primary goal of thymic T-cell development is to generate an anticipatory T-cell repertoire of the greatest possible size to ensure efficient immune responses to foreign pathogens while precluding the development of autoimmunity. However, LAT^G135D^ CD8 T cells exhibited restricted cell fates, with skewing toward effector cells, indicative of worse cell fate plasticity during immune responses. Such an imbalance in the ability of LAT^G135D^ T cells to adopt various cell fates highlights the evolutionary fitness conferred by proper regulation of the TCR proofreading step. Future experiments are needed to examine the potential impact of the LAT^G135D^ alteration upon CD8 T-cell memory responses and to explore the hypothesis that slow kinetic proofreading in mammals has created T cells that utilize TCR ligand discrimination to identify optimal agonistic signals, thereby retaining considerable plasticity to generate effector responses and form memory cells while retaining proper sensitivity to weak ligands.

T-cell ligand discrimination is particularly sensitive to the phosphorylation kinetics of LAT Y136 (among all Zap-70 substrates), plausibly because it is the sole tyrosine associated with PLC-γ1 interaction and function. Our data, together with complementary results by others on mice with a mutation conferring a Tyr136Phe alteration in LAT (LAT^Y136F^)^16–19^—in which the recruitment and activation of PLC-γ1 are completely disrupted—provide an opportunity to identify the divergent signaling pathways propagated through different tyrosine residues in LAT. Selective disruption or enhancement of LAT Y136–PLC-γ1 signaling has only a modest effect on ERK signaling, allowing LAT^Y136F^ to retain certain LAT signalosome functions^19^ and LAT^G135D^ to specifically tune PLC-γ1-specific signal transduction. Thus, we postulate that the slow phosphorylation associated with PLC-γ1 signaling and fast phosphorylation associated with Grb2/SOS and ERK/MAPK signaling enable TCR self- and nonself-discrimination to establish pathway specificity within LAT signalosomes. The LAT–PLC-γ1–calcium–NFAT pathway is, by default, the last to be activated after TCR engagement. Our single-amino acid modification of LAT uniquely facilitates NFAT signaling relative to other TCR downstream pathways, consequently altering T-cell development and homeostasis. As a result, LAT Y136 signal augmentation in LAT^G135D^ mice results in superior induction of thymic negative selection and peripheral T-cell anergy—the tolerance mechanisms that LAT^Y136F^ T cells fail to engage.

Interestingly, we did not observe spontaneous upregulation of coinhibitory receptors, including PD-1, in LAT^G135D^ mice, perhaps because the coinhibitory receptors are more distal in the TCR pathway and may be most important to prevent immunopathology once an immune response has been initiated. However, signaling domains that either compete with the LAT signalosome or interact with LAT downstream signaling are upregulated. For example, upregulation of CD5 and CD6 may cause assembly of their respective signalosomes, leading to competition with LAT signalosomes for interacting proteins^45,46^. In addition, molecules that directly inhibit signaling downstream of the LAT–PLC-γ1 pathway, such as DGK-ζ^47^, are also upregulated. Together, our data reveal the elements of downstream TCR signaling that are specifically dependent on LAT–PLC-γ1–calcium–NFAT signals. These results show that the importance of the LAT Y136–PLC-γ1 pathway lies in its duality: it equips T cells with enhanced responsiveness and sensitivity while priming them for tolerance induction. The LAT–PLC-γ1 pathway requires conditions to be just right, as both signaling deficiency and hyperactivity can lead to immunodeficiency. Overall, our study demonstrates an important physiological role of a kinetic proofreading step and unmasks the importance of coordinating signal specificity within LAT signalosomes to reinforce proper TCR ligand discrimination.

## Methods

### Experimental models

#### Mice

The C57BL/6, CD45.1^+^C57BL/6, Nur77–eGFP, MHC-II^–/–^, TAP^–/–^b2m^–/–^ or TCR Cα^–/–^ mice were housed in specific pathogen-free facilities at the University of California, San Francisco, University of Utah or Technical University of Munich. Mice were treated according to protocols that were approved by the University of California, San Francisco veterinary committees (A.W.), University of Utah veterinary committees (W.-L.L.) or Regierung von Oberbayern (D.Z.) and in accordance with National Institutes of Health guidelines or the requirements of EU Directive 2010/63/EU (Annex III, Part B, Table 1.1.). The mouse housing conditions were between 20 and 26 °C with 30–70% humidity (for mouse housing at the University of California, San Francisco), between 21 and 23 °C with 20–30% humidity (for the mouse housing at the University of Utah) or at ~22 °C and ~55% relative humidity (for the mouse housing at the Technical University of Munich). A 12 h light/12 h dark cycle was used. LAT^G135D^ mice were generated via electroporation of guide RNA and Cas9 messenger RNA. In brief, Cas9 protein (40 μM; QB3 MacroLab, University of California, Berkeley) and LAT guide RNAs (80 μM; either sgRNA#1 or sgRNA#2; Integrated DNA Technologies), along with 1–3 μl 200-base pair homology-directed repair template at 1 μg μl^−1^ concentration (Integrated DNA Technologies), were mixed and electroporated into C57BL/6 zygotes. The sequence of the homology-directed repair template was 5′–GAGGCTGGACCTGTCCAGGTCGTGTTAACTCTCCTTTCTCACAGAGCCAGCCTGTAAGAATGTGGATGCAGATGAGGATGAAGACGACTATCCCAACGATTACCTGTGAGTGGGTAGAGGGGAGGTGACCGTGGAAGTTGTGTGCCCTTTATCAACTTCTCGTTCCTTCCTTTCTTCCAGAGTGGTGCTGCCTGACAGTA-3′. The sgRNA #1 binding sequence was CCCAACGGCTACCTGTGAGT. The sgRNA #2 binding sequence was CCCACTCACAGGTAGCCGTT. A total of two founder lines with the desired LAT^G135D^ knockin were identified through a screen for PCR genotyping and confirmed via sequencing of cloned PCR products. These lines were backcrossed for at least four generations onto the C57BL/6 genetic background before they were used in the experiments presented here. Initial experiments showed no differences in the results between the progeny from the two founders.

Both males and females were used in the studies unless specifically specified. Specific ages of the mice have also been detailed, either in the Results section or in the figure captions.

#### Cell lines

The mouse T lymphoblast EL4 cell line was maintained in Dulbecco’s Modified Eagle’s Medium culture medium supplemented with 10% fetal bovine serum (FBS), 2 mM glutamine and 0.5 mg ml^−1^ of the aminoglycoside geneticin (G418; Santa Cruz Biotechnology).

### Antibodies

The following antibodies were used (all at a dilution of 1:400 unless specifically specified below) and are listed as antibody name (clone; catalog number(s); manufacturer; dilution): BV421 or BUV395 rat anti-mouse CD4 (clone GK1.5; 562891 or 563790; BD Biosciences); AF647 rat anti-mouse CD4 (clone APC; 557681; BD Biosciences); APC-Cy7 or BUV805 rat anti-mouse CD8α (clone 53-6.7; 557654 (1:100 dilution for staining) or 612898; BD Biosciences); BV711 rat anti-mouse CD8α (clone 53-6.7; 100748; BioLegend); AF647 rat anti-mouse CD8β (clone H35-17.2; 567661; BD Biosciences); BV711 rat anti-mouse CD62L (clone MEL-14; 568286; BD Biosciences); BV510 mouse anti-mouse H-2K^b^ (clone AF6-88.5; 742859; BD Biosciences); BV786, PE or PE-Cy7 hamster anti-mouse CD69 (clone H1.2F3; 564683, 553237 or 561930; BD Biosciences); PerCP-Cy5.5 hamster anti-mouse TCRβ (clone H57-597; 109228; BioLegend; 1:100 dilution for staining); PE-CF594 hamster anti-mouse TCRβ (clone H57-597; 562841; BD Biosciences; 1:800 dilution for staining); BUV395 rabbit anti-active caspase-3 (clone C92-605; 564095; BD Biosciences; 1:100 dilution for staining); PE, BUV737 or BUV805 rat anti-mouse CD5 (clone 53-7.3; 553022, 612809 or 741910; BD Biosciences); PE or APC rat anti-mouse CD5 (clone 53-7.3; 100608 or 100626; BioLegend); AF647 rat anti-mouse CCR9 (clone 9B1; 129710; BioLegend; 1:100 dilution for staining); PE-CF94 rat anti-mouse CCR7 (clone 4B12; 563596; BD Biosciences; 1:200 dilution for staining); BV421 or APC rat anti-mouse CD44 (clone IM7; 563970 or 559250; BD Biosciences); BUV395 rat anti-mouse CD6 (clone J90-462; 747534; BD Biosciences); purified rabbit anti-mouse DGKζ (clone EPR22040-80 or EPR22040-72; ab239081 or ab239080; Abcam); eFluor 660 rat anti-mouse TOX (clone TXRX10; 50-6502-82; Thermo Fisher/eBioscience; 1:200 dilution for staining); PerCP-Cy5.5 rat anti-mouse PD-1 (clone 29F.1A12; 135208; BioLegend); PerCP-eF710 Armenian hamster anti-mouse PD-1 (clone J43; 46-9985-82; Thermo Fisher/eBiosciences; 1:100 dilution for staining); BUV395 rat anti-mouse LAG-3 (clone C9B7W; 745693; BD Biosciences; 1:100 dilution for staining); BV510 mouse anti-mouse TIM-3 (clone 5D12/TIM-3; 747625; BD Biosciences; 1:100 dilution for staining); APC-R700 mouse anti-mouse TIGIT (clone 1G9; 565474; BD Biosciences; 1:100 dilution for staining); PE rat anti-mouse VISTA (clone MIH64; 566270; BD Biosciences; 1:300 dilution for staining); BV605 mouse anti-mouse CD73 (clone TY/11.8; 752734; BD Biosciences); PE-Cy7 rat anti-mouse FR4 (clone 12A5; 125012; BioLegend); AF488 or PE rat anti-mouse Foxp3 (clone MF-14; 126406 or 126404; BioLegend; 1:100 dilution for staining); BV605 or APC-Cy7 rat anti-mouse CD25 (clone PC61; 563061 or 557658; BD Biosciences); BV510 rat anti-mouse TNF (clone MF6-XT22; 563386; BD Biosciences); PE-Cy7 rat anti-mouse TNF (clone MF6-XT22; 25-7321-82; Thermo Fisher/eBiosciences); PE-Cy7 rat anti-mouse IFNγ (clone XMG1.2; 505826; BioLegend); PE rat anti-mouse IFNγ (clone XMG1.2; 12-7311-82; Thermo Fisher/eBiosciences); BV421 rat anti-mouse IL-2 (clone JES6-5H4; 554428; BD Biosciences); PE or AF647 mouse anti-Stat5 pY694 (clone 47/Stat5(pY694); 612567 or 612599; BD Biosciences); FITC mouse anti-mouse CD45.2 (clone 104; MCD45201; Thermo Fisher/eBiosciences); PE-Cy7 hamster anti-mouse KLRG1 (clone 2F1; 25-5893-82; Thermo Fisher/eBiosciences); APC rat anti-mouse CD127 (clone A7R34; 17-1271-82; Thermo Fisher/eBiosciences); eFluor 450 rat anti-mouse Ki-67 (clone SolA15; 17-5698-82; Thermo Fisher/eBiosciences); APC rat anti-mouse Ki-67 (clone 16A8; 652406; BioLegend); PE mouse anti-mouse granzyme B (clone QA16A02; 372208; BioLegend); PE mouse anti-TCF1 (clone S33-966; 564217; BD Biosciences); AF647 or PE rabbit anti-mouse/human NFAT (clone D43B1; 14201 or 14335; Cell Signaling Technology; 1:100 dilution for staining); PerCP-eFluor 710 mouse anti-mouse Nur77 (clone 12.14; 46-5965-82; Thermo Fisher/eBiosciences; 1:100 dilution for staining); PE mouse anti-NFκB (clone L8F6; 9460; Cell Signaling Technology; 1:100 dilution for staining); PE-Cy7 rat anti-mouse Egr-2 (clone erongr2; 25-6691-82; Thermo Fisher/eBiosciences; 1:100 dilution for staining); PE mouse anti-EOMES (566749; clone X4-83; BD Biosciences; 1:100 dilution for staining); rabbit polyclonal anti-mouse/human phospho-LAT (Tyr191) (20172; Cell Signaling Technology; 1:1,000 dilution for immunoblot analysis); rabbit polyclonal anti-mouse/human phospho-LAT (Tyr132) (44-224; Thermo Fisher Scientific; 1:1,000 dilution for immunoblot analysis); rabbit anti-mouse/human LAT (clone E3U6J; E3U6J; Cell Signaling Technology; 1:1,000 dilution for immunoblot analysis); mouse anti-alpha tubulin (clone B-5-1-2; T5168; Sigma–Aldrich; 1:1,000 dilution for immunoblot analysis); rabbit polyclonal anti-mouse/human Zap-70 (Tyr493)/Syk (Tyr526) (2704; Cell Signaling Technology; 1:1,000 dilution for immunoblot analysis); rabbit anti-mouse/human PLC-γ1 (Tyr783) (clone D6M9S; 14008; Cell Signaling Technology; 1:1,000 dilution for immunoblot analysis); mouse anti-mouse/human PLC-γ1 (clones B-2-5, B-6-4, B-20-3, D-7-3 and E-9-4; 05-163; MilliporeSigma; 1:1,000 dilution for immunoblot analysis); rabbit monoclonal anti-mouse/human phospho‐p44/42 MAPK (Thr202/Tyr204) (clone 197G2; 4377; Cell Signaling Technology; 1:1,000 dilution for immunoblot analysis); rabbit anti-mouse/human Bim (clone C34C5; 2933; Cell Signaling Technology; 1:1,000 dilution for immunoblot analysis); rabbit anti-mouse/human cleaved caspase-3 (Asp175) (clone 5A1E; 9664; Cell Signaling Technology; 1:1,000 dilution for immunoblot analysis); hamster anti-mouse CD28 (clone 37.51; 70-0281-U500 Tonbo Biosciences); biotin Armenian hamster anti-mouse CD3ε (clone 145-2C11; 30-0031-U500; Tonbo Biosciences); donkey anti-mouse IgG (715-035-151; Jackson ImmunoResearch; 1:10,000 dilution for immunoblot analysis); goat anti-mouse IgG light chain (115-035-174; Jackson ImmunoResearch; 1:10,000 dilution for immunoblot analysis); donkey anti-rabbit IgG (711-035-152; Jackson ImmunoResearch; 1:10,000 dilution for immunoblot analysis); and mouse anti-rabbit IgG light chain (211-032-171; Jackson ImmunoResearch; 1:10,000 for immunoblot analysis).

### Analysis of thymic clonal deletion at the population level

Thymic clonal deletion was characterized by analysis of the expression levels of CCR7, CCR9 and cleaved caspase-3 (ref. ^31^). In brief, thymi were harvested and processed as quickly as possible to avoid nonspecific apoptosis. A total of 1 × 10^7^ thymocytes were first stained with anti-CCR7 and/or anti-CCR9 antibody for 30 min at 37 °C, followed by staining of the surface markers CD5, TCRβ, CD4 and CD8. Thymocytes were then fixed with 4% freshly prepared paraformaldehyde (BeanTown Chemical) and washed with Perm/Wash buffer (BD Biosciences) twice. Next, thymocytes were stained with anti-cleaved caspase-3 (Asp175) at a 1:100 dilution for 30 min at room temperature. Cells were washed with Perm/Wash buffer and analyzed on an LSRFortessa system (BD Biosciences).

### Analysis of intracellular calcium

Preselection thymocytes were obtained by depleting CD53^+^ cell populations. In brief, thymocytes were prepared and stained with 50 μl anti-CD53 antibody per 2 × 10^8^ cells for 15 min on ice. After cells were washed twice with MCAS running buffer (Dulbecco’s phosphate-buffered saline (PBS) supplemented with 0.5% bovine serum albumin (BSA) and 2 mM ethylenediaminetetraacetic acid (EDTA)), they were further stained with 20 μl anti-rat IgM biotin (Jackson ImmunoResearch) per 2 × 10^8^ cells for 15 min on ice. After they were washed twice, CD53^–^ thymocytes were enriched through a column, collected and counted. The purity of isolated preselection CD53^–^ thymocytes was confirmed by the staining of CD4, CD8, CD5, CD69 and TCRβ. The purity of cells was consistently >95%. Preselection CD53^–^ thymocytes were then loaded with 1 μM of the calcium indicator dye Indo-1 (Thermo Fisher Scientific) and 0.02% Pluronic F-127 (Thermo Fisher Scientific) at 37 °C in Roswell Park Memorial Institute (RPMI) medium supplemented with 5% FBS for 30 min, washed twice with PBS and then labeled with a 1:100 dilution of biotinylated anti-mouse CD3ε antibody on ice for 30 min. Cells were then used to perform flow cytometry-based calcium assays. Indo-1 cell-associated fluorescence was first recorded for 30 s to obtain a baseline and then monitored after additions of streptavidin (10 μg ml^−1^) at the 30th second. The calcium responses were recorded for a total of 5 min. Similarly, CD4 periphery T cells were enriched using a homemade purification kit (A.W. laboratory). CD4 T cells were then stained with the surface markers CD4, CD44, CD62L, CD73 and FR4 on ice for 30 min. Cells were washed twice and then subjected to the same experimental set up to perform experiments of calcium responses on an LSRFortessa.

### In vitro T-cell stimulation assays

Naive or anergic mouse OT-I CD8 cells were sorted and cocultured at a 5:1 ratio with OVA or APL-pulsed TCR Cα^–/–^ splenocytes overnight over a titrated dose of antigens, as indicated in the figures, then plated at a concentration of 10^5^ cells in round-bottomed 96-well plates in 200 μl complete RPMI media containing 10% fetal calf serum (FCS), 1× nonessential amino acids, 2 mM glutamine, 1 mM sodium pyruvate, 0.05% gentamicin and 50 μM 2-mercaptoethanol. The next day, cells were examined for their upregulation of CD69 and CD25, proliferation or cytokine production. For the proliferation assays, cells were labeled with 5 μM CellTrace Violet dye (Thermo Fisher Scientific)—a fluorescent dyes able to track proliferation—in PBS at 37 °C for 20 min in the dark. Labeled cells were incubated with complete culture medium at 37 °C for another 5 min, washed twice and cultured with peptide-pulsed splenocytes for 4 d. The proliferation ability of the cells was then analyzed by flow cytometry. As for the intracellular cytokine assays, cells were stimulated with OVA or APL-pulsed TCR Cα^–/–^ splenocytes overnight. The next day, 2 μM monensin (BioLegend) was added 4 h before the harvest of the cells. Cells were first stained with the surface markers CD4, CD8, CD25, CD69, CD73 and FR4, followed by fixation and permeabilization in Cytofix/Cytoperm (BD Biosciences) solution for 10–20 min on ice. The intracellular cytokines IFNγ, TNF and IL-2 were stained in Cytofix/Cytoperm (BD Biosciences) solution for 30 min on ice.

### FTOC

The matings of wild-type or LAT^G135D^ OT-I.*Rag1*^–/–^.*Tap1*^–/–^ mice were set up and timed to obtain embryos at an approximate gestational age of 16–17 d. The thymic lobes were harvested and cultured at 37 °C in RPMI medium supplemented with 10% FCS, 1× nonessential amino acids, 2 mM glutamine, 1 mM sodium pyruvate, 0.05% gentamicin and 50 μM 2-mercaptoethanol. After 4 d of incubation, the thymic lobes were harvested and stained for flow cytometry analysis.

### Flow cytometry-based analysis of nuclear translocation of transcriptional factor

The base nuclei isolation and staining protocol was adapted from a previous LAT^G135D^ OT-I T cells were sorted and stimulated with OVA-pulsed TCR Cα^–/–^ splenocytes for the indicated number of hours. After stimulation, cells were harvested and spun down at 300*g* and 4 °C and the pellets were immediately resuspended with 250 μl ice-cold PBS containing 320 mM sucrose (pH 7.4), 10 mM HEPES (Life Technologies), 8 mM MgCl_2_, 1× Roche EDTA-free cOmplete Protease Inhibitor (MilliporeSigma) and 0.1% (vol/vol) Triton X-100 (MilliporeSigma). After 15 min on ice, the plate was spun at 2,000*g* and 4 °C for 10 min. This was followed by two 250 μl washes with ice-cold PBS containing 320 mM sucrose (pH 7.4), 10 mM HEPES (Life Technologies), 8 mM MgCl_2_ and 1× Roche EDTA-free cOmplete Protease Inhibitor (MilliporeSigma) at 2,000*g* and 4 °C. After the final wash, pellets were fixed in 4% paraformaldehyde (electron microscopy grade; Electron Microscopy Sciences) and nuclei were rested on ice for 30 min for fixation, followed by two washes, resuspension in 1× PBS with 2% FBS and centrifugation at 1,000*g* to sufficiently pellet the nuclei. Nuclei were kept at 4 °C until flow cytometry analysis.

### ELISA for serum anti-dsDNA

Serum was harvested from blood collected by lateral tail vein sampling or cardiac puncture postmortem. The serum anti-dsDNA titer was measured with a commercial ELISA kit, per the manufacturer’s instructions (Alpha Diagnostic International). In brief, sera were added to plates coated with dsDNA. Anti-dsDNA titer was detected with anti-IgG-HRP. ELISA plates were developed and the absorbance was measured.

### Antinuclear antibodies

Serum antinuclear antibodies (ANAs) were detected with a NOVA Lite HEp-2 ANA Substrate Slide, per the manufacturer’s instructions, except for using FITC-conjugated donkey anti-mouse IgG secondary antibody. Images were captured with a Zeiss Axio Imager M2 widefield fluorescence microscope. Images were processed with ZEN pro (Zeiss). To measure the titer, the serum was serially diluted twofold from 1:40 to 1:1,280. HEp-2 ANA slides were stained with diluted serum. Images were read by a rheumatologist in a blinded manner and the titer was determined as the detectable lowest dilution of each sample. Notably, we selected only females to establish the aging cohort, to control for sex as a biological variable.

### Immunoblot analysis

CD53^–^ or DP thymocytes were enriched as above. Thymocytes were washed with PBS and resuspended at 5 × 10^6^ cells per ml and rested for 30 min at 37 °C. Cells were labeled with biotinylated anti-CD3ε (clone 2C11) at the indicated concentration. Cells were left unstimulated or stimulated with the addition of streptavidin over time, as described for each experiment. Cells were lysed by directly adding 10% NP-40 lysis buffer to a final concentration of 1% NP-40 (containing inhibitors of 2 mM NaVO_4_, 10 mM NaF, 5 mM EDTA, 2 mM phenylmethylsulfonyl fluoride, 10 μg ml^−1^ aprotinin, 1 μg ml^−1^ pepstatin and 1 μg ml^−1^ leupeptin). Lysates were placed on ice and centrifuged at 13,000*g* to pellet cell debris. Supernatants were run on NuPAGE 4–12% Bis-Tris Protein Gels (Thermo Fisher Scientific) and transferred to polyvinylidene difluoride membranes. Membranes were blocked using Tris-buffered saline with 0.1% Tween 20 detergent buffer containing 3% BSA and then probed with primary antibodies, as described for each experiment, overnight at 4 °C. The following day, blots were rinsed and incubated with horseradish peroxidase-conjugated secondary antibodies (Jackson ImmunoResearch). Blots were developed using a chemiluminescent substrate and a Bio-Rad ChemiDoc imaging system (Bio-Rad).

### 2D *K*_d_ affinity measurement

The relative 2D affinities of naive OT-I H-2K^b^ CD8 T cells were measured using the previously characterized 2D-MP. Briefly, red blood cells were coated with biotin-LC-NHS (BioVision) and streptavidin (Thermo Fisher Scientific), together with either the biotinylated pMHC wild-type OVA (SIINFEKL WT (OVA)) or an OVA APL monomer (SIIQFERL (Q4R7), SIITFEKL (T4), SIIQFEKL (Q4), SIIQFEHL (Q4H7), SIIVFEKL (V4), SIIGFEKL (G4) or SIIRFEKL (R4)) and mouse β2-microglobulin (National Institutes of Health Tetramer Core Facility). To specifically investigate the TCR:pMHC interaction, monomers were generated with a H-2K^b^ a3 domain with a human HLA-A2 a3 domain to mitigate CD8 coreceptor binding. A red blood cell coated with the monomer of interest and a T cell of interest were mounted onto opposing micropipettes. An electronically controlled piezoelectric actuator repeated a T-cell contact and separation cycle with the pMHC-coated red blood cell 50 times while keeping the contact area (*A*_c_) and time (*t*) constant. Following retraction of the cell, binding of the TCR:pMHC was observed as a distention of the red blood cell membrane using an inverted microscope, allowing for quantification of the adhesion frequency (*P*_a_) at equilibrium. Surface pMHC (ml) and TCR (mr) densities were determined by flow cytometry using anti-TRCβ PE antibody (clone H57-597; BD Biosciences) and PE anti-mouse β2-microglobulin antibody (clone A16041A; BioLegend), both at saturating concentrations, along with BD Quantibrite PE beads for standardization (BD Biosciences). The relative 2D affinity was calculated using the following equation: 2D affinity (*A*_c_*K*_a_) = −ln[1 − *P*_a_(2s)]*m*_r_*m*_l_. The ‘*m*_r_’ indicates TCR surface density and ‘*m*_l_’ indicates ligand (pMHC) surface density. The *P*_a_(2s) means the adhesion frequency (or probability of adhesion) at 2 second (s) contact time.

### *Listeria* infection

CD8 T cells were harvested from wild-type or LAT^G135D^ OT-I.*Rag1*^–/–^ mice and enriched through negative selection using A.W. laboratory-customized biotinylated antibody cocktails (a mixture of anti-CD4 or anti-CD8 together with anti-CD19, anti-B220, anti-CD11b, anti-CD11c, anti-DX5, anti-TER119 and anti-CD24) and magnetic bead-mediated negative selection (Anti-Biotin Miltenyi iBeads; Miltenyi Biotec). Enriched CD8 T cells were then stained with anti-CD62L and anti-CD44 and the naive cell population was sorted based on CD62L^+^CD44^–^. Cells were then frozen in BloodStor 100 Freezing Medium for Hematopoietic Cells (STEMCELL Technologies) and shipped to Germany on dry ice. Some 2 × 10^4^ naive OT-I.*Rag1*^–/–^ CD8 T cells were transferred into C57BL/6 mice 1 d before infection. Recombinant *L. monocytogenes* strains stably expressing native OVA (SIINFEKL (N4)) or the APL (SIIVFEKL (V4)) were previously described^35^. Mice were infected intravenously with 1,000 colony-forming units of *Listeria* in log phase. Recombinant vesicular stomatitis virus (Indiana strain) expressing SIINFEKL (N4) was grown and titrated on baby hamster kidney cells. Mice were infected intravenously with 2 × 10^6^ plaque-forming units.

### Surface and intracellular antibody staining of murine T cells

Single-cell suspensions of splenocytes were obtained by mashing whole spleens through a 100-μm nylon cell strainer (BD Falcon). Red blood cells were lysed with a hypotonic ammonium–chloride–potassium buffer. Blocking of unspecific antibody binding was achieved with 2.4G2 (BioXCell). Surface staining was performed for 30 min at 4 °C in PBS supplemented with 2% FCS (Sigma–Aldrich) and 0.01% azide (Sigma–Aldrich) using the following antibodies: anti-CD45.2-FITC (clone 104); anti-KLRG1-PE-Cy7 (clone 2F1); anti-CD127-APC (clone A7R34); anti-PD-1-PerCP-eF710 (clone J43); and anti-CD8-BV711 (clone 53-6.7). Cells were fixed in PBS containing 2% formaldehyde for 15 min, then washed and resuspended in FACS buffer.

For intracellular cytokine staining, ~3 × 10^6^ splenocytes were first stimulated with peptide at 37 °C for a total of 5 h. After 30 min, 7 μM brefeldin A was added. Cells were fixed in PBS with 2% formaldehyde and permeabilized in PBS containing 0.25% saponin and 0.25% BSA (Perm Buffer; BD Biosciences). Cells were then stained for 40 min at 4 °C with anti-IFNγ-PE (clone XMG1.2) and anti-TNF-PE-Cy7 (clone MP6-XT22) antibodies in Perm Buffer. Intracellular cytokine staining of granzyme B (clone QA16A02) was conducted according to the same protocol without peptide stimulation. Intracellular transcription factor staining was performed using the Foxp3/Transcription Factor Staining kit. Cells were stained with anti-TCF1-PE (clone S33-966) and anti-Ki-67-eF450 (clone SolA15) antibodies. Flow cytometry measurements were performed using an LSRFortessa flow cytometer (BD Biosciences). All data were analyzed using FlowJo (TreeStar).

### Quantification and statistical analysis

Statistical analysis was applied to technical replicates or biologically independent mice for each experiment. All experiments described in this study have been performed at least twice and the exact numbers of independent experiments with similar results are indicated in the figure captions. All statistical analyses of experiments were performed using nonparametric, two-tailed Mann–Whitney *U*-tests. Image Lab (Bio-Rad) version 5.2.1 built 11 was used to acquire immunoblot data and BD FACSDiva version 8.0.1 software was used for flow cytometry. FlowJo version 9.9.3 or 10.8.1 was used for flow cytometry data analysis. SnapGene software version 4.0.8 was used to analyze DNA sequences or Sanger sequence data. GraphPad Prism 7 or 9 software (GraphPad Software) was used for data analysis and representation. All bar graphs show means with overlaid scatter dots or error bars (indicating s.d.) to show the distribution of the data, as indicated in each figure caption. *P* values for comparisons are provided as exact values or as *P* < 0.0001. 95% confidence levels were used to determine statistically significant *P* values. No statistical methods were used to predetermine sample sizes but our sample sizes were similar to those reported in previous publications. The data met the assumptions of the statistical tests used. Data distributions (individual data points) have been shown in all figures when applicable. Data distributions were assumed to be normal but this was not formally tested. No randomization was used in the experiments. In the animal experiments, age-matched animals were allocated based on their genotypes. In cell stimulation experiments, cells with the same genotype were pooled together and equally allocated into different groups before treatments. Data collection and analysis were not performed blind to the conditions of the experiments, except for the autoantibody ELISA and staining analysis and the hematoxylin and eosin staining-based immunopathology analysis. No data points or animals were excluded from the analysis.

### Reporting summary

Further information on research design is available in the Nature Portfolio Reporting Summary linked to this article.

## Online content

Any methods, additional references, Nature Portfolio reporting summaries, source data, extended data, supplementary information, acknowledgements, peer review information; details of author contributions and competing interests; and statements of data and code availability are available at 10.1038/s41590-023-01444-x.

## Supplementary information

Supplementary Information Supplementary Fig. 1.

Reporting Summary

## Data Availability

All of the data that support the findings of the present study are available from the corresponding authors upon request. Source data are provided with this paper.

## Source data

Source Data Fig. 1 Statistical source data.
Source Data Fig. 2 Statistical source data.
Source Data Fig. 2 Unprocessed western blots.
Source Data Fig. 3 Statistical source data.
Source Data Fig. 4 Statistical source data.
Source Data Fig. 5 Statistical source data.
Source Data Fig. 6 Statistical source data.
Source Data Fig. 7 Statistical source data.
Source Data Fig. 8 Statistical source data.
Source Data Extended Data Fig. 2 Statistical source data.
Source Data Extended Data Fig. 3 Statistical source data.
Source Data Extended Data Fig. 3 Unprocessed western blots.
Source Data Extended Data Fig. 4 Statistical source data.
Source Data Extended Data Fig. 5 Statistical source data.
Source Data Extended Data Fig. 6 Statistical source data.
Source Data Extended Data Fig. 7 Statistical source data.
Source Data Extended Data Fig. 8 Statistical source data.
Source Data Extended Data Fig. 9 Statistical source data.
Source Data Extended Data Fig. 10 Statistical source data.

## References

[1] Huseby ES, Teixeiro E (2022). The perception and response of T cells to a changing environment are based on the law of initial value. Sci. Signal..

[2] Hogquist KA, Jameson SC (2014). The self-obsession of T cells: how TCR signaling thresholds affect fate ‘decisions’ and effector function. Nat. Immunol..

[3] Juang J (2010). Peptide–MHC heterodimers show that thymic positive selection requires a more restricted set of self-peptides than negative selection. J. Exp. Med..

[4] Pettmann J (2021). The discriminatory power of the T cell receptor. eLife.

[5] Stepanek O (2014). Coreceptor scanning by the T cell receptor provides a mechanism for T cell tolerance. Cell.

[6] McKeithan TW (1995). Kinetic proofreading in T-cell receptor signal transduction. Proc. Natl Acad. Sci. USA.

[7] Lo WL, Weiss A (2021). Adapting T cell receptor ligand discrimination capability via LAT. Front. Immunol..

[8] Ganti RS (2020). How the T cell signaling network processes information to discriminate between self and agonist ligands. Proc. Natl Acad. Sci. USA.

[9] Yousefi OS (2019). Optogenetic control shows that kinetic proofreading regulates the activity of the T cell receptor. eLife.

[10] Tischer DK, Weiner OD (2019). Light-based tuning of ligand half-life supports kinetic proofreading model of T cell signaling. eLife.

[11] Lo WL (2019). Slow phosphorylation of a tyrosine residue in LAT optimizes T cell ligand discrimination. Nat. Immunol..

[12] Balagopalan L, Kortum RL, Coussens NP, Barr VA, Samelson LE (2015). The linker for activation of T cells (LAT) signaling hub: from signaling complexes to microclusters. J. Biol. Chem..

[13] Balagopalan L, Coussens NP, Sherman E, Samelson LE, Sommers CL (2010). The LAT story: a tale of cooperativity, coordination, and choreography. Cold Spring Harb. Perspect. Biol..

[14] Fu G (2010). Phospholipase Cγ1 is essential for T cell development, activation, and tolerance. J. Exp. Med..

[15] Rao A, Luo C, Hogan PG (1997). Transcription factors of the NFAT family: regulation and function. Annu. Rev. Immunol..

[16] Sommers CL (2002). A LAT mutation that inhibits T cell development yet induces lymphoproliferation. Science.

[17] Aguado E (2002). Induction of T helper type 2 immunity by a point mutation in the LAT adaptor. Science.

[18] Mingueneau M (2009). Loss of the LAT adaptor converts antigen-responsive T cells into pathogenic effectors that function independently of the T cell receptor. Immunity.

[19] Sommers CL (2005). Mutation of the phospholipase C-γ1-binding site of LAT affects both positive and negative thymocyte selection. J. Exp. Med..

[20] Myers DR, Norlin E, Vercoulen Y, Roose JP (2019). Active tonic mTORC1 signals shape baseline translation in naive T cells. Cell Rep..

[21] Khan O (2019). TOX transcriptionally and epigenetically programs CD8^+^ T cell exhaustion. Nature.

[22] Martinez GJ (2015). The transcription factor NFAT promotes exhaustion of activated CD8^+^ T cells. Immunity.

[23] Shah NH (2016). An electrostatic selection mechanism controls sequential kinase signaling downstream of the T cell receptor. eLife.

[24] Stadinski BD (2019). A temporal thymic selection switch and ligand binding kinetics constrain neonatal Foxp3^+^ T_reg_ cell development. Nat. Immunol..

[25] Stritesky GL (2013). Murine thymic selection quantified using a unique method to capture deleted T cells. Proc. Natl Acad. Sci. USA.

[26] Baldwin TA, Hogquist KA (2007). Transcriptional analysis of clonal deletion in vivo. J. Immunol..

[27] Winoto A, Littman DR (2002). Nuclear hormone receptors in T lymphocytes. Cell.

[28] Zikherman J, Parameswaran R, Weiss A (2012). Endogenous antigen tunes the responsiveness of naive B cells but not T cells. Nature.

[29] Dzhagalov IL, Chen KG, Herzmark P, Robey EA (2013). Elimination of self-reactive T cells in the thymus: a timeline for negative selection. PLoS Biol..

[30] Bouillet P (2002). BH3-only Bcl-2 family member Bim is required for apoptosis of autoreactive thymocytes. Nature.

[31] Breed ER, Watanabe M, Hogquist KA (2019). Measuring thymic clonal deletion at the population level. J. Immunol..

[32] McDonald BD, Bunker JJ, Erickson SA, Oh-Hora M, Bendelac A (2015). Crossreactive αβ T cell receptors are the predominant targets of thymocyte negative selection. Immunity.

[33] Drobek A (2018). Strong homeostatic TCR signals induce formation of self-tolerant virtual memory CD8 T cells. EMBO J..

[34] Miller CH (2020). Eomes identifies thymic precursors of self-specific memory-phenotype CD8^+^ T cells. Nat. Immunol..

[35] Zehn D, Lee SY, Bevan MJ (2009). Complete but curtailed T-cell response to very low-affinity antigen. Nature.

[36] Hogquist KA (1994). T cell receptor antagonist peptides induce positive selection. Cell.

[37] Hogquist KA (2001). Assays of thymic selection. Fetal thymus organ culture and in vitro thymocyte dulling assay. Methods Mol. Biol..

[38] Gallagher MP, Conley JM, Berg LJ (2018). Peptide antigen concentration modulates digital NFAT1 activation in primary mouse naive CD8^+^ T cells as measured by flow cytometry of isolated cell nuclei. ImmunoHorizons.

[39] Jennings E (2020). Nr4a1 and Nr4a3 reporter mice are differentially sensitive to T cell receptor signal strength and duration. Cell Rep..

[40] Fathman CG, Lineberry NB (2007). Molecular mechanisms of CD4^+^ T-cell anergy. Nat. Rev. Immunol..

[41] Schmitz ML, Krappmann D (2006). Controlling NF-κB activation in T cells by costimulatory receptors. Cell Death Differ..

[42] Beverly B, Kang SM, Lenardo MJ, Schwartz RH (1992). Reversal of in vitro T cell clonal anergy by IL-2 stimulation. Int. Immunol..

[43] Chappert P, Schwartz RH (2010). Induction of T cell anergy: integration of environmental cues and infectious tolerance. Curr. Opin. Immunol..

[44] Schwartz RH (2003). T cell anergy. Annu. Rev. Immunol..

[45] Mori D (2021). The T cell CD6 receptor operates a multitask signalosome with opposite functions in T cell activation. J. Exp. Med..

[46] Voisinne G (2022). Kinetic proofreading through the multi-step activation of the ZAP70 kinase underlies early T cell ligand discrimination. Nat. Immunol..

[47] Shifrut E (2018). Genome-wide CRISPR screens in primary human T cells reveal key regulators of immune function. Cell.

