# Supplementary material for: A single-amino acid substitution in the adaptor LAT accelerates TCR proofreading kinetics and alters T-cell selection, maintenance and function
Source: Nat Immunol. 2023 Mar 13;24(4):676–89. doi: 10.1038/s41590-023-01444-x (PMC10063449; doi:10.1038/s41590-023-01444-x)
Supplement: Supplementary file 1 — Supplementary Fig. 1. [file 41590_2023_1444_MOESM1_ESM.pdf]

nature immunology

Article

https://doi.org/10.1038/s41590-023-01444-x

A single-amino acid substitution in the adaptor LAT accelerates TCR proofreading kinetics and alters T-cell selection, maintenance and function

In the format provided by the authors and unedited

Supplementary Figure 1

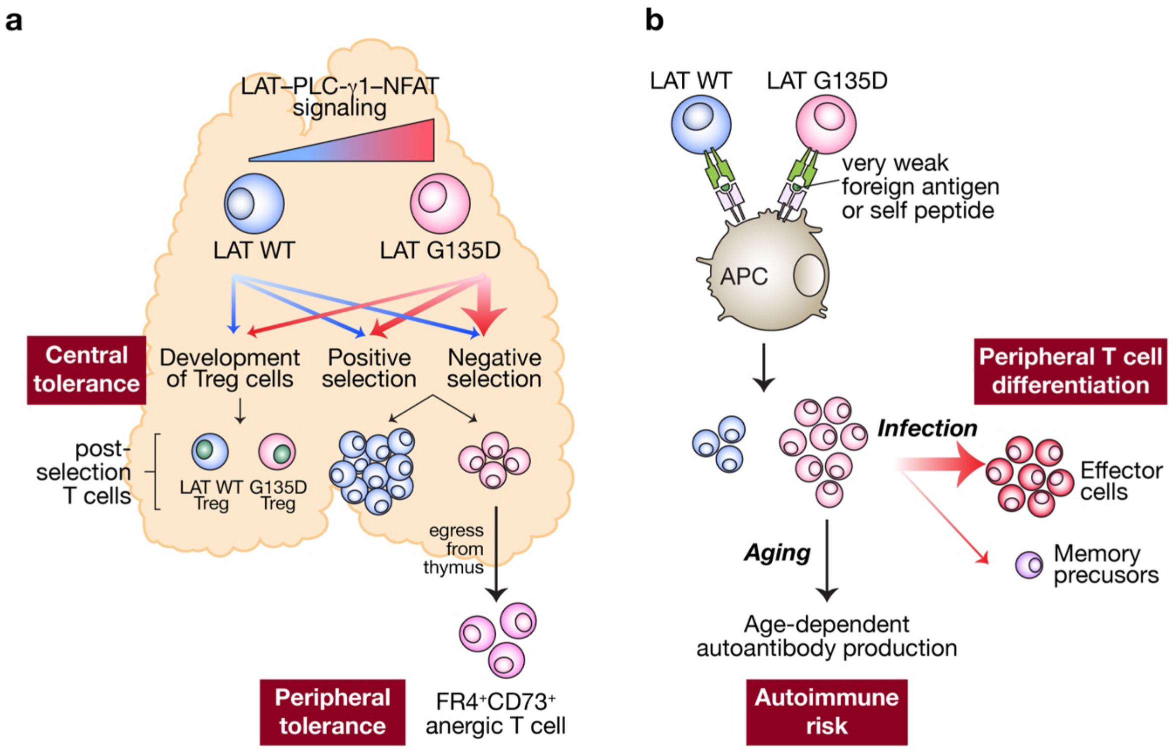

Illustration of the physiological importance and pathological consequences of disrupting kinetic proofreading by accelerating the phosphorylation of LAT Y136.

a. Expression of G135D LAT shortens the molecular time delay required for the TCR to proofread pMHC signals, thereby endowing T cells with enhanced sensitivity to self-pMHC molecules. The G135D LAT mutation specifically augments the LAT–PLC-g1–NFAT pathway, with minimal effects on other pathways. The enhanced self-reactivity of G135D LAT T cells triggers excessive thymic negative selection and promotes T cell anergy. G135D T cells exhibit enhanced engagement of central and peripheral tolerance with no obvious impact on Treg cell frequencies and homeostasis. The thickness of the colored arrows indicates the degree of the effect.

b. Despite their enhanced engagement of central and peripheral tolerance mechanisms, when G135D mice age, they develop high titers of autoantibodies (ANA and anti-dsDNA) and severe chronic inflammation in the gut mucosa. During *Listeria* infection, G135D LAT T cells are able to proliferate more robustly than wild-type T cells to very weak foreign ligands. However, G135D LAT also promotes the terminal differentiation of antigen-specific CD8 T cells and impairs the formation of memory precursors in response to strong TCR stimuli. Thus, the double-edged sword of G135D LAT cuts two ways: G135D LAT-expressing T cells display heightened responses to pathogens; however, LAT G135D-mediated alterations may predispose to impaired formation of memory precursors during infection and elevated risk of autoimmune diseases with age.
