# Supplementary material for: A single-amino acid substitution in the adaptor LAT accelerates TCR proofreading kinetics and alters T-cell selection, maintenance and function
Source: Nat Immunol. 2023 Mar 13;24(4):676–89. doi: 10.1038/s41590-023-01444-x (PMC10063449; doi:10.1038/s41590-023-01444-x)
Supplement: Supplementary file 2 — Reporting Summary [file 41590_2023_1444_MOESM2_ESM.pdf]

Corresponding author(s): Arthur Weiss; Dietmar Zehn; Wan-Lin Lo

Last updated by author(s): Jan 22, 2023

Reporting Summary

Nature Portfolio wishes to improve the reproducibility of the work that we publish. This form provides structure for consistency and transparency in reporting. For further information on Nature Portfolio policies, see our Editorial Policies and the Editorial Policy Checklist.

Statistics

For all statistical analyses, confirm that the following items are present in the figure legend, table legend, main text, or Methods section.

n/a	Confirmed	
☐	☒	The exact sample size (*n*) for each experimental group/condition, given as a discrete number and unit of measurement
☐	☒	A statement on whether measurements were taken from distinct samples or whether the same sample was measured repeatedly
☐	☒	The statistical test(s) used AND whether they are one- or two-sided<br>*Only common tests should be described solely by name; describe more complex techniques in the Methods section.*
☒	☐	A description of all covariates tested
☐	☒	A description of any assumptions or corrections, such as tests of normality and adjustment for multiple comparisons
☐	☒	A full description of the statistical parameters including central tendency (e.g. means) or other basic estimates (e.g. regression coefficient) AND variation (e.g. standard deviation) or associated estimates of uncertainty (e.g. confidence intervals)
☐	☒	For null hypothesis testing, the test statistic (e.g. *F*, *t*, *r*) with confidence intervals, effect sizes, degrees of freedom and *P* value noted<br>*Give P values as exact values whenever suitable.*
☒	☐	For Bayesian analysis, information on the choice of priors and Markov chain Monte Carlo settings
☒	☐	For hierarchical and complex designs, identification of the appropriate level for tests and full reporting of outcomes
☒	☐	Estimates of effect sizes (e.g. Cohen's *d*, Pearson's *r*), indicating how they were calculated

Our web collection on statistics for biologists contains articles on many of the points above.

Software and code

Policy information about availability of computer code

Data collection	Image Lab (Bio-Rad) V5.2.1 built 11 was used to acquire immunoblot data; BD FACSDiva v8.0.1 software was used for flow cytometry.
Data analysis	Prism (GraphPad) Version 7 or 9 was used to data analysis; FlowJo, Version 9.9.3 or Version 10.8.1 was used for flow cytometry data analysis. SnapGene software v4.0.8 was used to analyze DNA sequences or Sanger sequence data.

For manuscripts utilizing custom algorithms or software that are central to the research but not yet described in published literature, software must be made available to editors and reviewers. We strongly encourage code deposition in a community repository (e.g. GitHub). See the Nature Portfolio guidelines for submitting code & software for further information.

Data

Policy information about availability of data

All manuscripts must include a data availability statement. This statement should provide the following information, where applicable:

- Accession codes, unique identifiers, or web links for publicly available datasets
- A description of any restrictions on data availability
- For clinical datasets or third party data, please ensure that the statement adheres to our policy

The primary data for analysis of all figures and supplementary figures are available upon request. All corresponding authors are committed to the transparent reporting of methods and data, and to the open distribution of published data and reagents. To ensure that these ideals will be upheld, all three research groups maintained a thorough record and backup of all information generated over the course of these projects, and make these resources available upon request.

Human research participants

Policy information about studies involving human research participants and Sex and Gender in Research.

Reporting on sex and gender	The study did not involve human research participants.
Population characteristics	The study did not involve human research participants so the information was not collected.
Recruitment	The study did not involve human research participants so there were no participants recruited.
Ethics oversight	The study did not involve human research participants so there is no protocol required to be approved.

Note that full information on the approval of the study protocol must also be provided in the manuscript.

Field-specific reporting

Please select the one below that is the best fit for your research. If you are not sure, read the appropriate sections before making your selection.

☒ Life sciences ☐ Behavioural & social sciences ☐ Ecological, evolutionary & environmental sciences

For a reference copy of the document with all sections, see nature.com/documents/nr-reporting-summary-flat.pdf

Life sciences study design

All studies must disclose on these points even when the disclosure is negative.

Sample size	An initial sample size used in each experiment is determined empirically based on pilot or routine experiments. In each experiment, at least 3 mice in each genotype group were used to ensure proper biological replicates. Reproducibility among multiple independent experiments was used to determine the proper sample size and statistic power are achieved. All presented data have been at least independently performed twice.
Data exclusions	Results show all data points collected from the experiments described in the manuscripts.
Replication	Data presented here were consistent and repetitively observed. All data were repeated for at least two independent experiments and related details were included in the Methods and Figure Legends in the main Article.
Randomization	Mice were sex and age matched, littermates were used whenever possible. Mice were then allocated into experimental groups according to their genotypes.
Blinding	Histological analysis and anti-dsDNA titer determination were performed through double-blind set up. The bar-coded experimental set up allowed us to examine WT and mutant samples under the same experimental conditions. Data collection and analysis were not performed blind to the conditions of the experiments, except for the autoantibody ELISA and staining analysis, and the H&E staining-based immunopathology analysis. No data points or animals were excluded from the analysis.

Reporting for specific materials, systems and methods

We require information from authors about some types of materials, experimental systems and methods used in many studies. Here, indicate whether each material, system or method listed is relevant to your study. If you are not sure if a list item applies to your research, read the appropriate section before selecting a response.

Materials & experimental systems

n/a	Involved in the study	
☐	☒	Antibodies
☐	☒	Eukaryotic cell lines
☒	☐	Palaeontology and archaeology
☐	☒	Animals and other organisms
☒	☐	Clinical data
☒	☐	Dual use research of concern

Methods

n/a	Involved in the study	
☒	☐	ChIP-seq
☐	☒	Flow cytometry
☒	☐	MRI-based neuroimaging

Antibodies

Antibodies used	All antibody staining were used as 1:400 dilution unless specifically specified below. BV421, or BUV395 rat anti-mouse CD4 (clone GK1.5; BD Biosciences; catalog #562891; #563790); AF647 rat anti-mouse CD4 (clone APC, BD Biosciences; catalog #557681); APC-Cy7 or, BUV805 , or BV711 rat anti-mouse CD8 (clone 53-6.7; BD Biosciences or Biolegend; catalog #557654 [1:100 dilution for staining]; #612898)); BV711 rat anti-mouse CD8 (clone 53-6.7; Biolegend; catalog #100748); APC AF647 rat anti-mouse CD8 (clone H35-17.2; BD Biosiences; catalog #567661); BV711 rat anti-mouse CD62L (clone MEL-14; BD Biosciences; catalog #568286); BV510 mouse anti-mouse H-2Kb (clone AF6-88.5; BD Biosciences; catalog #742859); BV786, PE, or PE-Cy7 hamster anti-mouse CD69 (clone H1.2F3; BD Biosciences; catalog #564683; # 553237; #561930); PerCP-Cy5.5 hamster anti-mouse TCR (clone H57-597; BioLegend; catalog #109228; 1:100 dilution for staining); PE-CF594 hamster anti-mouse TCR (clone H57-597; BD Biosciences; catalog #562841 [1:800 dilution for staining]); BUV395 rabbit anti-active caspase-3 (clone C92-605; BD Biosciences; catalog # 564095; 1:100 dilution for staining); PE, BUV3737 or BUV 805 rat anti-mouse CD5 (clone 53-7.3; BD Biosciences; catalog #553022; #612809; #741910); PE or APC rat anti-mouse CD5 (clone 53-7.3; BioLegend; catalog #100608; #100626); AF647 rat anti-mouse CCR9 (clone 9B1; BioLegend; catalog #129710; 1:100 dilution for staining); PE-CF94 rat anti-mouse CCR7 (clone 4B12; BD Biosciences; catalog #563596; 1:200 dilution for staining); BV421 or APC rat anti-mouse CD44 (clone IM7; BD Biosciences; catalog #563970; #559250); BUV395 rat anti-mouse CD6 (clone J90-462; BD Biosciences; #747534); purified rabbit anti-mouse DGK (clone EPR22040-80 or EPR22040-72; abcam; catalog #ab239081; #ab239080); eFlour 660 rat anti-mouse TOX (clone TXRX10; Thermo Fisher/eBioscience; catalog # 50-6502-82; 1:200 dilution for staining); PerCP or PerCP-Cy5.5 rat anti-mouse PD-1 (clone 29F.1A12; BioLegend; catalog #135208); PerCP-eF710 Armenian hamster anti-mouse PD-1 (clone J43; Thermo Fisher/eBiosciences; catalog #46-9985-82; 1:100 dilution for staining); BUV395 rat anti-mouse LAG-3 (clone C9B7W; BD Biosciences; catalog #745693; 1:100 dilution for staining); BV510 mouse anti-mouse TIM-3 (clone 5D12/TIM-3; BD Biosciences; catalog #747625; 1:100 dilution for staining); APC-R700 mouse anti-mouse TIGIT (clone 1G9; BD Biosciences; catalog #565474; 1:100 dilution for staining); PE rat anti-mouse VISTA (clone MIH643; BD Biosciences; catalog #566270; 1:300 dilution for staining); BV605 mouse anti-mouse CD73 (clone TY/11.8; BD Biosciences; catalog #752734); PE-Cy7 rat anti-mouse FR4 (clone 12A5; BioLegend; catalog #125012); AF488 or PE rat anti-mouse Foxp3 (clone MF-14; BioLegend; catalog #126406; #126404; 1:100 dilution for staining); BV605 or APC-Cy7 rat anti-mouse CD25 (clone PC61; BD Biosciences; catalog #563061; #557658); BV510 rat anti-mouse TNF (clone MF6-XT22; BD Biosciences; catalog #563386); PE-Cy7 rat anti-mouse TNF (clone MF6-XT22; Thermo Fisher/eBiosciences; catalog #25-7321-82); PE-Cy7 rat anti-mouse IFN- (clone XMG1.2; BioLegend; catalog # 505826); PE rat anti-mouse IFN- (clone XMG1.2; Thermo Fisher/eBiosciences; catalog #12-7311-82); BV421 rat anti-mouse IL-2 (clone JES6-5H4; BD Biosciences; catalog #554428); PE or AF647 mouse anti-Stat5 pY694 (clone 47/Stat5(pY694); BD Biosciences; catalog #612567; #612599); FITC mouse anti-mouse CD45.2 (clone 104; Thermo Fisher/eBiosciences; catalog #MCD45201); PE-Cy7 hamster anti-mouse KLRG1 (clone 2F1; Thermo Fisher/eBiosciences; catalog #25-5893-82); APC rat anti-mouse CD127 (clone A7R34; Thermo Fisher/eBiosciences; catalog #17-1271-82); eFlour 450 rat anti-mouse Ki-67 (clone SolA15; Thermo Fisher/eBiosciences; catalog #17-5698-82); APC rat anti-mouse Ki-67 (clone 16A8; BioLegend; catalog #652406); PE mouse anti-mouse granzyme B (clone QA16A02; BioLegends; catalog #372208); PE mouse anti-TCF1 (clone S33-966; BD Biosciences; catalog #564217); AF647 or PE rabbit anti-mouse/human NFAT (clone D43B1; Cell Signaling Technology; catalog #14201; #14335; 1:100 dilution for staining); PerCP-eFlour 710 mouse anti-mouse Nur77 (clone 12.14; Thermo Fisher/eBiosciences; catalog #46-5965-82; 1:100 dilution for staining); PE mouse anti-NFB (clone L8F6; Cell Signaling Technology; catalog # 9460; 1:100 dilution for staining); PE-Cy7 rat anti-mouse Egr-2 (clone erongr2; Thermo Fisher/eBiosciences; catalog #25-6691-82; 1:100 dilution for staining); PE mouse anti-EOMES (clone X4-83; BD Biosciences; catalog # 566749; 1:100 dilution for staining); rabbit polyclonal anti-mouse/human phospho-LAT (Tyr191) (Cell Signaling Technology; catalog # 20172; 1:1000 dilution for immunoblot analysis); rabbit polyclonal anti-mouse/human phospho-LAT (Tyr132) (Thermo Fisher Scientific; catalog #44-224; 1:1000 dilution for immunoblot analysis); rabbit polyclonal anti-mouse/human LAT (clone E3U6J; Cell Signaling Technology; catalog #E3U6J; 1:1000 dilution for immunoblot analysis); mouse anti-alpha tubulin (clone B-5-1-2; Sigma-Aldrich; catalog #T5168; 1:1000 dilution for immunoblot analysis); rabbit polyclonal anti-mouse/human Zap-70 (Tyr493)/Syk (Tyr526) (Cell Signaling Technology; catalog #2704; 1:1000 dilution for immunoblot analysis); rabbit anti-mouse/human PLC-1 (Tyr783) (clone D6M9S; Cell Signaling Technology; catalog #14008; 1:1000 dilution for immunoblot analysis); mouse anti-mouse/ human PLC-1 (clone B-2-5, B-6-4, B-20-3, D-7-3, and E-9-4; Millipore Sigma; catalog #05-163; 1:1000 dilution for immunoblot analysis); rabbit monoclonal anti-mouse/human phopspho-p44/42 MAPK (Thr202/Tyr204) (clone 197G2; Cell Signaling Technology; catalog #4377; 1:1000 dilution for immunoblot analysis); rabbit anti-mouse/human Bim (clone C34C5; Cell Signaling Technology; catalog #2933; 1:1000 dilution for immunoblot analysis); rabbit anti-mouse/human cleaved caspase-3 (Asp175) (clone 5A1E; Cell Signaling Technology; catalog #9664; 1:1000 dilution for immunoblot analysis); hamster anti-mouse CD28 (clone 37.51; Tonbo Biosciences; catalog #70-0281-U500); biotin Armenian hamster anti-mouse CD3 (clone 145-2C11; Tonbo Biosciences; catalog #30-0031-U500). Donkey anti–mouse IgG (Jackson ImmunoResearch; catalog #715-035-151; 1:10000 dilution for immunoblot anlaysis), goat anti–mouse IgG light chain (catalog #115-035-174; Jackson ImmunoResearch; 1:10000 dilution for immunoblot analysis), donkey anti–rabbit IgG (catalog #711-035-152; Jackson ImmunoResearch; 1:10000 dilution for immunoblot analysis) and mouse anti–rabbit IgG light chain (catalog #211-032-171; Jackson ImmunoResearch; 1:10000 for immunoblot analysis).
Validation	All the antibodies are from commercial sources and have been validated by the vendors. Validation data are available on the manufacturer's website. The LAT phospho-Y132, phospho-Y191 antibodies were validated using the Y132F mutant or Y191F mutant expressing Jurkat cells and J.LAT deficient Jurkat cells and had been published before. All other validation statements could be on the manufacturer's website.

Eukaryotic cell lines

Policy information about cell lines and Sex and Gender in Research

Cell line source(s)	The mouse T lymphoblast EL4 cell line was originally obtained from ATCC #TIB-39 by the Weiss lab and maintained by UCSF Tissue Culture Core facility
Authentication	Certificate of analysis was provided by ATCC at the time of purchasing. Other than this, no specific authentication was performed specifically.
Mycoplasma contamination	The EL4 line has been tested for mycoplasma negative in past years.

Commonly misidentified lines (See ICLAC register)	The EL4 cell line was not among the misidentified cell lines published from the most recent version 11.0 of the database.

Animals and other research organisms

Policy information about studies involving animals; ARRIVE guidelines recommended for reporting animal research, and Sex and Gender in Research

Laboratory animals	The C57BL/6, CD45.1+C57BL/6, Nur77-eGFP, MHC-II–/–, TAP–/–b2m–/¬–, or TCR C[illegible]–/– mice were housed in the specific pathogen-free facilities at the University of California, San Francisco, University of Utah, or Technical University of Munich. Mice were treated according to protocols that were approved by University of California, San Francisco veterinary committees (to A.W.), by University of Utah veterinary committees (to W.-L. L), or by Technical University of Munich animal care ethics committee (to D.Z.), and are in accordance with NIH guidelines or the requirements of EU Directive 2010/63/EU (Annex III, Part B, Table 1.1.). The mouse housing conditions are between 68-79 oF with 30-70% humidity (for mouse housing at the University of California, San Francisco), or between 70-74 oF with 20-30% humidity (for the mouse housing at the University of Utah), or at approx. 22 oC and approx. 55% relative humidity (for the mouse housing at the Technical University of Munich). A 12 hour light/12 hour dark cycle is used.
Wild animals	The study did not involve wild animals.
Reporting on sex	Both male and female mice were used for the study unless specifically indicated. For the aging experiments, only female mice were included.
Field-collected samples	The study did not involve samples collected from the field.
Ethics oversight	Animals were maintained in accordance with University of Utah Animal Care and Use Committee protocols, University of California, or approved by San Francisco veterinary committees, or by Technical University of Munich animal care ethics committee, and are in accordance with NIH guidelines.

Note that full information on the approval of the study protocol must also be provided in the manuscript.

Flow Cytometry

Plots

Confirm that:

☒ The axis labels state the marker and fluorochrome used (e.g. CD4-FITC).

☒ The axis scales are clearly visible. Include numbers along axes only for bottom left plot of group (a 'group' is an analysis of identical markers).

☒ All plots are contour plots with outliers or pseudocolor plots.

☒ A numerical value for number of cells or percentage (with statistics) is provided.

Methodology

Sample preparation	Spleens and lymph node single cell suspensions were prepared by gently tweezing samples in cold PBS buffer containing 0.5% FBS and 0.2% EDTA. Samples were then counted. Up to 0.5 million T cells were washed with PBS + 0.5% FBS and 0.2% EDTA and labeled with indicated antibody. Samples were then labeled with antibody cocktails of interests for 30 min ~ 1 hr on ice, followed by 2x wash and resuspended in 100 ul PBS + 0.5% FBS + 0.5% EDTA and analyzed on flow cytometry. For intracellular staining, cells were fixed by 4% PFA or using Foxp3 staining kit. Cells were permeabilized and stained with indicated antibody.
Instrument	BD LSR Fortessa was used to collect flow cytometry data. BD FACSAria III was used for cell sorting.
Software	BD FACSDiva v8.0.1 software was used to collect samples. FlowJo v9.9.3 or v10 was used to analyze flow cytometry data.
Cell population abundance	Sorted naive CD25-CD44lowCD62Lhi OT-I CD8 population was about 98% purity.
Gating strategy	For all data, viable lymphocytes were gated by FCS-A/SSC-A, as well as live/dead marker for some experiments, and also gate on single cells using FCS-A/FCS-H. Positive populations were determined by staining as well as unstained controls. Gating strategy is shown in extended data.

☒ Tick this box to confirm that a figure exemplifying the gating strategy is provided in the Supplementary Information.
