# Supplementary figures and images for: A single-amino acid substitution in the adaptor LAT accelerates TCR proofreading kinetics and alters T-cell selection, maintenance and function
Source: Nat Immunol. 2023 Mar 13;24(4):676–89. doi: 10.1038/s41590-023-01444-x (PMC10063449; doi:10.1038/s41590-023-01444-x)

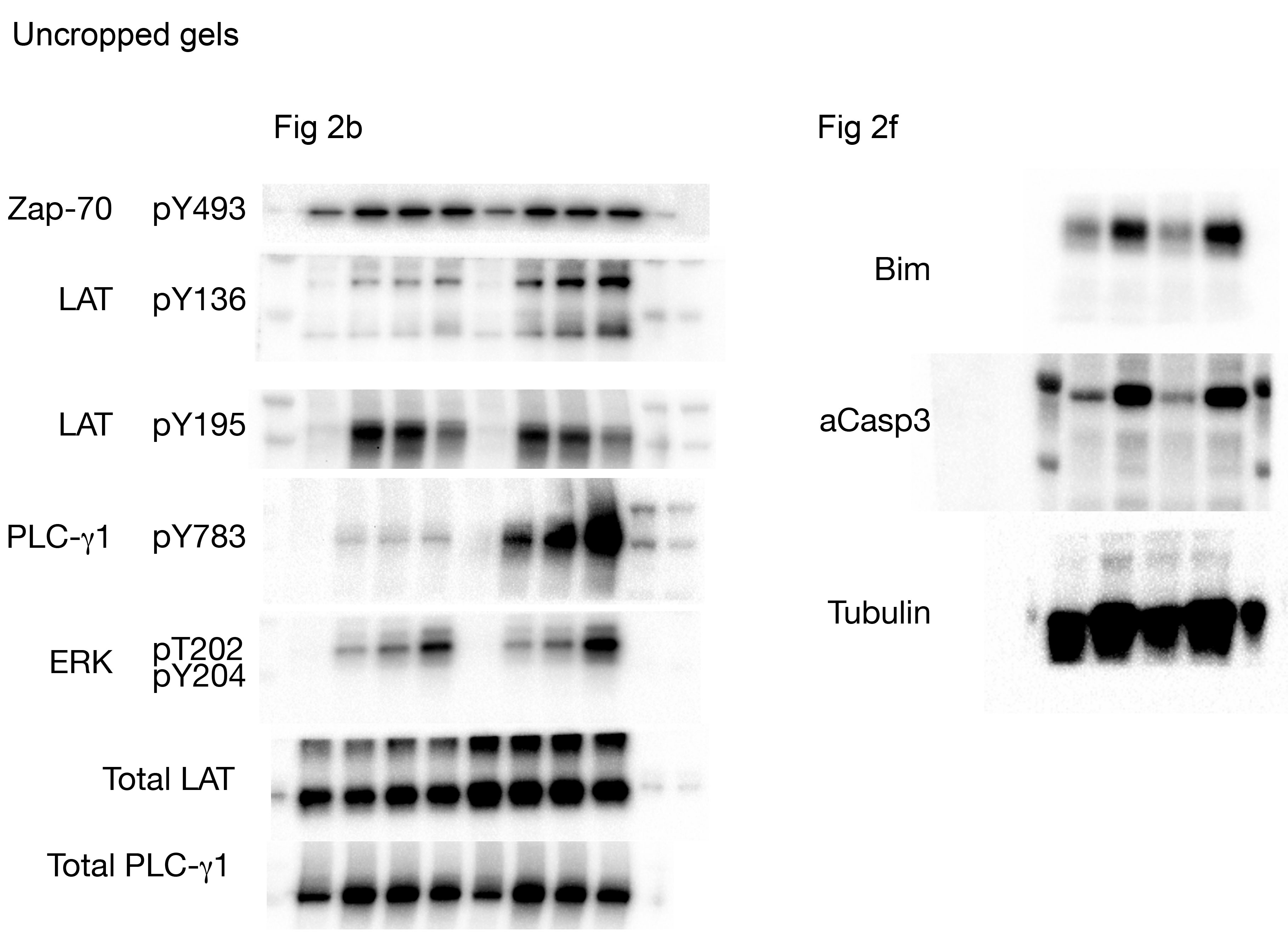

Supplement: Source Data Fig. 2 — Unprocessed western blots. [file 41590_2023_1444_MOESM5_ESM.jpg]

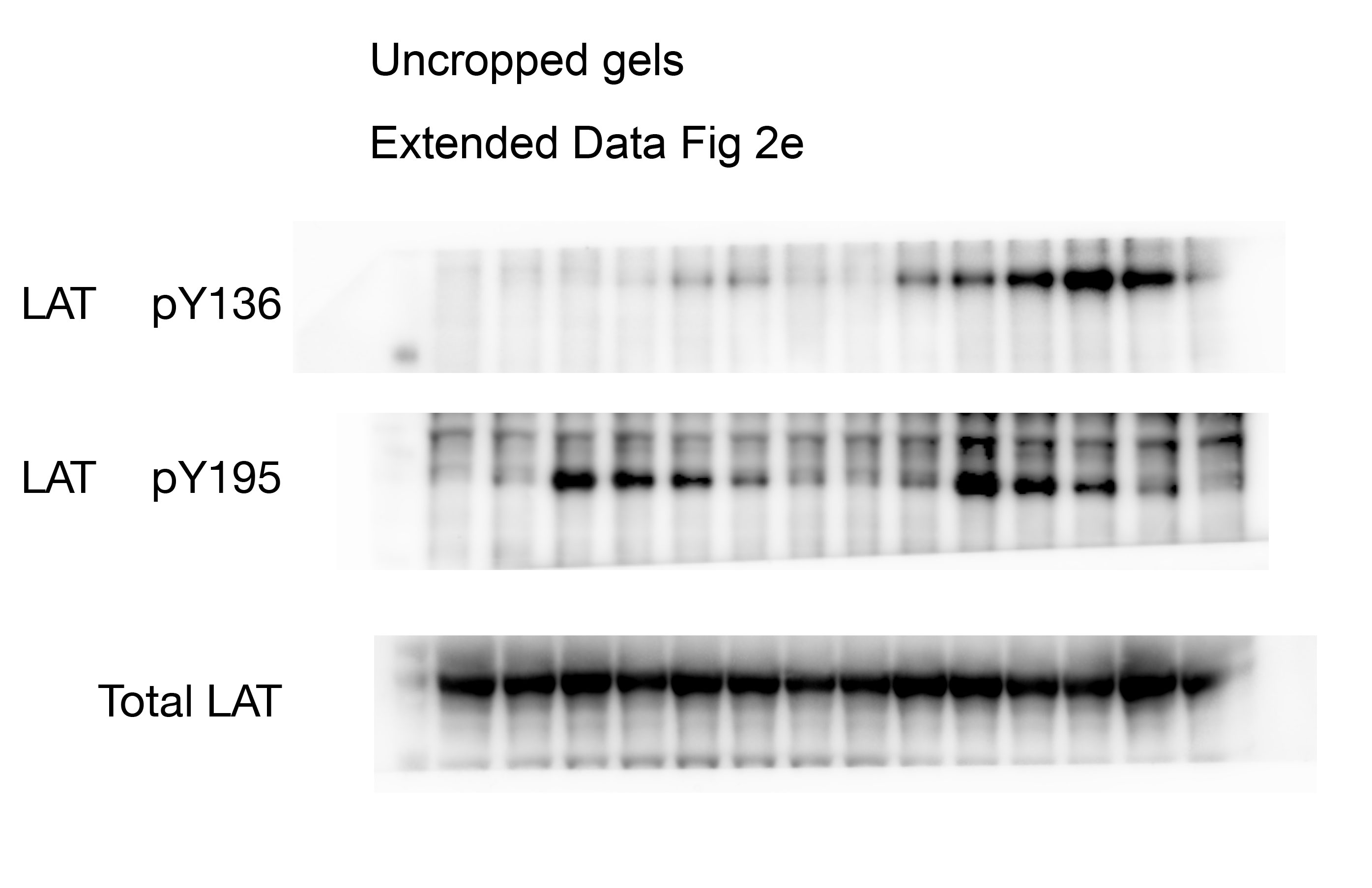

Supplement: Source Data Extended Data Fig. 3 — Unprocessed western blots. [file 41590_2023_1444_MOESM14_ESM.jpg]
